# Inter3D: Capture of TAD Reorganization Endows Variant Patterns of Gene Transcription

**DOI:** 10.1093/gpbjnl/qzae034

**Published:** 2024-05-08

**Authors:** Tianyi Ding, Shaliu Fu, Xiaoyu Zhang, Fan Yang, Jixing Zhang, Haowen Xu, Jiaqi Yang, Chaoqun Chen, Yibing Shi, Yiran Bai, Wannian Li, Xindi Chang, Shanjin Wang, Chao Zhang, Qi Liu, He Zhang

**Affiliations:** State Key Laboratory of Cardiology and Medical Innovation Center, Institute for Regenerative Medicine, Shanghai East Hospital, Frontier Science Research Center for Stem Cells, School of Life Science and Technology, Tongji University, Shanghai 200092, China; Jiangxi Province Key Laboratory of Organ Development and Epigenetics, Clinical Medical Research Center, Affiliated Hospital of Jinggangshan University, Medical Department of Jinggangshan University, Ji'an 343009, China; School of Life Science, Jinggangshan University, Ji'an 343009, China; State Key Laboratory of Cardiology and Medical Innovation Center, Institute for Regenerative Medicine, Shanghai East Hospital, Frontier Science Research Center for Stem Cells, School of Life Science and Technology, Tongji University, Shanghai 200092, China; State Key Laboratory of Cardiology and Medical Innovation Center, Institute for Regenerative Medicine, Shanghai East Hospital, Frontier Science Research Center for Stem Cells, School of Life Science and Technology, Tongji University, Shanghai 200092, China; Jiangxi Province Key Laboratory of Organ Development and Epigenetics, Clinical Medical Research Center, Affiliated Hospital of Jinggangshan University, Medical Department of Jinggangshan University, Ji'an 343009, China; School of Life Science, Jinggangshan University, Ji'an 343009, China; State Key Laboratory of Cardiology and Medical Innovation Center, Institute for Regenerative Medicine, Shanghai East Hospital, Frontier Science Research Center for Stem Cells, School of Life Science and Technology, Tongji University, Shanghai 200092, China; Jiangxi Province Key Laboratory of Organ Development and Epigenetics, Clinical Medical Research Center, Affiliated Hospital of Jinggangshan University, Medical Department of Jinggangshan University, Ji'an 343009, China; School of Life Science, Jinggangshan University, Ji'an 343009, China; State Key Laboratory of Cardiology and Medical Innovation Center, Institute for Regenerative Medicine, Shanghai East Hospital, Frontier Science Research Center for Stem Cells, School of Life Science and Technology, Tongji University, Shanghai 200092, China; Jiangxi Province Key Laboratory of Organ Development and Epigenetics, Clinical Medical Research Center, Affiliated Hospital of Jinggangshan University, Medical Department of Jinggangshan University, Ji'an 343009, China; School of Life Science, Jinggangshan University, Ji'an 343009, China; State Key Laboratory of Cardiology and Medical Innovation Center, Institute for Regenerative Medicine, Shanghai East Hospital, Frontier Science Research Center for Stem Cells, School of Life Science and Technology, Tongji University, Shanghai 200092, China; Jiangxi Province Key Laboratory of Organ Development and Epigenetics, Clinical Medical Research Center, Affiliated Hospital of Jinggangshan University, Medical Department of Jinggangshan University, Ji'an 343009, China; School of Life Science, Jinggangshan University, Ji'an 343009, China; State Key Laboratory of Cardiology and Medical Innovation Center, Institute for Regenerative Medicine, Shanghai East Hospital, Frontier Science Research Center for Stem Cells, School of Life Science and Technology, Tongji University, Shanghai 200092, China; Jiangxi Province Key Laboratory of Organ Development and Epigenetics, Clinical Medical Research Center, Affiliated Hospital of Jinggangshan University, Medical Department of Jinggangshan University, Ji'an 343009, China; School of Life Science, Jinggangshan University, Ji'an 343009, China; State Key Laboratory of Cardiology and Medical Innovation Center, Institute for Regenerative Medicine, Shanghai East Hospital, Frontier Science Research Center for Stem Cells, School of Life Science and Technology, Tongji University, Shanghai 200092, China; Jiangxi Province Key Laboratory of Organ Development and Epigenetics, Clinical Medical Research Center, Affiliated Hospital of Jinggangshan University, Medical Department of Jinggangshan University, Ji'an 343009, China; School of Life Science, Jinggangshan University, Ji'an 343009, China; State Key Laboratory of Cardiology and Medical Innovation Center, Institute for Regenerative Medicine, Shanghai East Hospital, Frontier Science Research Center for Stem Cells, School of Life Science and Technology, Tongji University, Shanghai 200092, China; Jiangxi Province Key Laboratory of Organ Development and Epigenetics, Clinical Medical Research Center, Affiliated Hospital of Jinggangshan University, Medical Department of Jinggangshan University, Ji'an 343009, China; School of Life Science, Jinggangshan University, Ji'an 343009, China; State Key Laboratory of Cardiology and Medical Innovation Center, Institute for Regenerative Medicine, Shanghai East Hospital, Frontier Science Research Center for Stem Cells, School of Life Science and Technology, Tongji University, Shanghai 200092, China; Jiangxi Province Key Laboratory of Organ Development and Epigenetics, Clinical Medical Research Center, Affiliated Hospital of Jinggangshan University, Medical Department of Jinggangshan University, Ji'an 343009, China; School of Life Science, Jinggangshan University, Ji'an 343009, China; State Key Laboratory of Cardiology and Medical Innovation Center, Institute for Regenerative Medicine, Shanghai East Hospital, Frontier Science Research Center for Stem Cells, School of Life Science and Technology, Tongji University, Shanghai 200092, China; State Key Laboratory of Cardiology and Medical Innovation Center, Institute for Regenerative Medicine, Shanghai East Hospital, Frontier Science Research Center for Stem Cells, School of Life Science and Technology, Tongji University, Shanghai 200092, China; State Key Laboratory of Cardiology and Medical Innovation Center, Institute for Regenerative Medicine, Shanghai East Hospital, Frontier Science Research Center for Stem Cells, School of Life Science and Technology, Tongji University, Shanghai 200092, China; State Key Laboratory of Cardiology and Medical Innovation Center, Institute for Regenerative Medicine, Shanghai East Hospital, Frontier Science Research Center for Stem Cells, School of Life Science and Technology, Tongji University, Shanghai 200092, China; State Key Laboratory of Cardiology and Medical Innovation Center, Institute for Regenerative Medicine, Shanghai East Hospital, Frontier Science Research Center for Stem Cells, School of Life Science and Technology, Tongji University, Shanghai 200092, China; Jiangxi Province Key Laboratory of Organ Development and Epigenetics, Clinical Medical Research Center, Affiliated Hospital of Jinggangshan University, Medical Department of Jinggangshan University, Ji'an 343009, China; School of Life Science, Jinggangshan University, Ji'an 343009, China; State Key Laboratory of Cardiology and Medical Innovation Center, Institute for Regenerative Medicine, Shanghai East Hospital, Frontier Science Research Center for Stem Cells, School of Life Science and Technology, Tongji University, Shanghai 200092, China; Jiangxi Province Key Laboratory of Organ Development and Epigenetics, Clinical Medical Research Center, Affiliated Hospital of Jinggangshan University, Medical Department of Jinggangshan University, Ji'an 343009, China; School of Life Science, Jinggangshan University, Ji'an 343009, China

**Keywords:** Topologically associating domain, Inter3D, Chromosomal loop, Gene transcription, Cancer

## Abstract

Topologically associating domain (TAD) reorganization commonly occurs in the cell nucleus and contributes to gene activation and inhibition through the separation or fusion of adjacent TADs. However, functional genes impacted by TAD alteration and the underlying mechanism of TAD reorganization regulating gene transcription remain to be fully elucidated. Here, we first developed a novel approach termed Inter3D to specifically identify genes regulated by TAD reorganization. Our study revealed that the segregation of TADs led to the disruption of intrachromosomal looping at the myosin light chain 12B (*MYL12B*) locus, via the meticulous reorganization of TADs mediating epigenomic landscapes within tumor cells, thereby exhibiting a significant correlation with the down-regulation of its transcriptional activity. Conversely, the fusion of TADs facilitated intrachromosomal interactions, suggesting a potential association with the activation of cytochrome P450 family 27 subfamily B member 1 (*CYP27B1*). Our study provides comprehensive insight into the capture of TAD rearrangement-mediated gene loci and moves toward understanding the functional role of TAD reorganization in gene transcription. The Inter3D pipeline developed in this study is freely available at https://github.com/bm2-lab/inter3D and https://ngdc.cncb.ac.cn/biocode/tool/BT7399.

## Introduction

The eukaryotic genome is evolutionarily conserved but also spatially highly plastic with cell specificity [1–3]. It is composed of multilevel structures encompassing chromosome territories, A/B compartments, topologically associating domains (TADs), and chromosomal loops [4,5]. At the macroscopic level, chromatin can be categorized into A and B compartments according to higher and lower transcriptional activities, respectively. The transformation between A/B compartments signifies the necessity for topological remodeling in the transcriptional regulation of genes [6]. At the micro level, dynamic chromosomal loops facilitate interactions between gene promoters and their respective regulatory elements, thereby influencing gene expression and different biological processes, including stem cell differentiation, reprogramming, immune response, learning and memory, and tumorigenesis [7–10]. Intriguingly, large local chromatin interaction domains between the macro-level and micro-level, known as TADs, serve as universal structural features and fundamental units within the genome and are conserved across diverse cell types in various mammalian species [11].

TADs play a pivotal role in facilitating the hierarchical folding of chromatin between different cellular groups and can be reconstituted by changing their own insulating boundaries, serving as a connecting link between the preceding and the following. TADs typically range in size from 200 kb to 1 Mb, and the TAD boundary is abundant in architectural elements, including CCCTC-binding factor (CTCF), cohesin, non-coding RNAs (ncRNAs), short interspersed nuclear elements (SINEs), and retrotransposons [11–13]. Loop extrusion-dominant boundaries (LEBs) and phase separation-dominant boundaries (PSBs) are two distinct types of TAD boundary [13–16]. These boundary insulators specifically bind to distinct motifs located at the boundaries of TADs, thus determining the positioning and extent of TADs. The deletion of boundary insulators has the potential to induce alterations in TAD organization at the cellular population level and promote interactions between genes residing within one TAD and *cis*-regulatory elements (CREs) located in another TAD across boundaries [17–21]. In vertebrates, TADs are formed by cohesin-mediated chromatin loop extrusion, and convergence-oriented CTCF sites determine TAD boundaries by interrupting this process [22]. However, in *Drosophila*, orthologs of CTCF do not function in TAD formation; instead, M1BP and BEAF-32, which have insulating properties, tend to play a more vital role in the formation of TAD boundaries [23–25]. Consequently, the comprehensive regulatory mechanism underlying TAD formation is not fully understood, and further studies are needed to identify the dominant factors involved in boundary insulation.

TADs play a crucial role in partitioning the genome into distinct regulatory units and can be subdivided into smaller units called subTADs. The functional association between distinct TADs/subTADs and DNA replication has been confirmed [26]. Individual TADs are relatively independent, and the interaction frequency within the TAD is significantly higher than that between adjacent TADs [11,27–29]. Nevertheless, TADs can further form larger units called metaTADs, within which intricate inter-TAD interactions can be interpreted as relatively straightforward, tree-like hierarchical arrangements, which is a characteristic that remains consistent across different cell types [30]. A recent study revealed that a newly developed proteolysis-targeting chimera (PROTAC) degrader can reduce switch/sucrose non-fermentable (SWI/SNF)-mediated enhancer accessibility and impede enhancer and promoter looping interactions, providing a promising TAD-based therapeutic approach for enhancer-addicted cancers [31]. Therefore, it is critical to identify these TAD alteration-mediated functional gene loci efficiently and further uncover the mechanisms underlying TAD reorganization in controlling DNA–DNA interactions and gene transcription.

In this study, we introduced a novel algorithm named Inter3D, which was designed with a specific focus on characterizing TAD reorganization and identifying differentially expressed genes (DEGs) influenced by alterations in TAD boundaries. Our investigation reveals that the identification of gene loci impacted by TAD rearrangements is crucial for understanding the functional significance of TAD reorganization in gene transcription. Furthermore, our findings shed light on the diverse patterns of gene transcription that drive TAD alterations.

## Method

### Capture of inter-TAD and intra-TAD interactions by Inter3D

**Figure 1:**
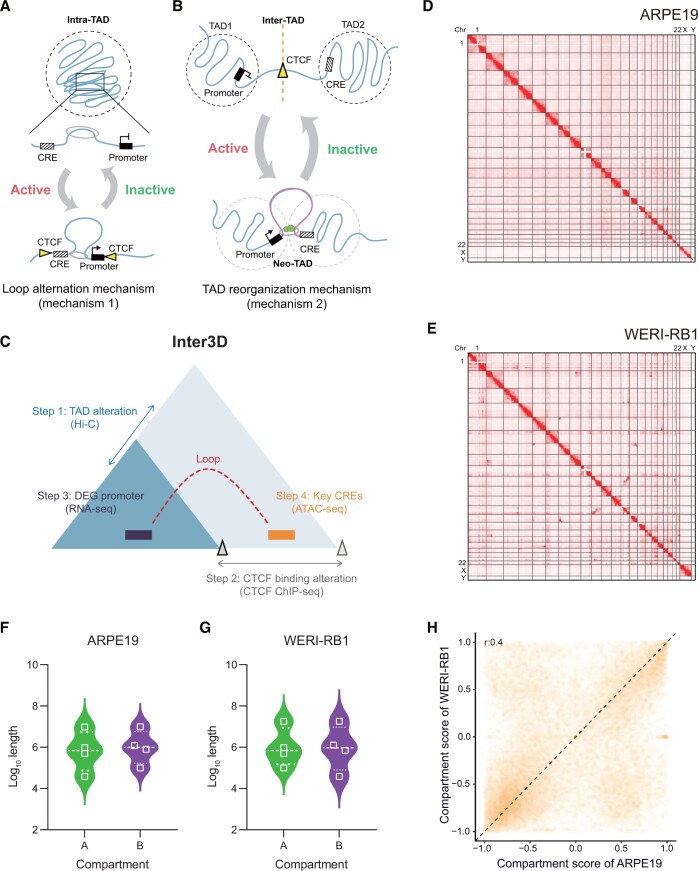
Capture of inter-TAD and intra-TAD contacts with the Inter3D pipeline **A**. and **B**. Two models of the 3D genome regulating gene transcription are loop alteration mechanism (mechanism 1) (A) and TAD reorganization mechanism (mechanism 2) (B). Yellow triangle represents CTCF; gray ring represents cohesin; and green circle represents other structural factors involved in looping in addition to CTCF mediating the TAD boundary. **C**. Model of TAD reorganization leading to alteration of CRE–gene chromosomal loops. Blue triangles represent TADs. Dotted arc represents nonexistent loop. The gray triangles indicate the CTCF-mediated TAD boundary. **D**. and **E**. Genome-wide chromatin interaction heatmaps of all chromosomes in ARPE19 (D) and WERI-RB1 (E) cells. **F**. and **G**. The length distribution of A/B compartments in ARPE19 (F) and WERI-RB1 (G) cells. **H**. Degree of A/B compartment overlap in ARPE19 and WERI-RB1 cells. TAD, topologically associating domain; CTCF, CCCTC-binding factor; CRE, *cis*-regulatory element; DEG, differentially expressed gene; Chr, chromosome; 3D, three-dimensional; Hi-C, high-throughput chromosome conformation capture; RNA-seq, RNA sequencing; ATAC-seq, assay for transposase-accessible chromatin with high-throughput sequencing; ChIP-seq, chromatin immunoprecipitation sequencing.

To address the aforementioned questions about TAD reorganization, we developed Inter3D, a user-friendly pipeline to identify three-dimensional (3D) interactome, by performing integrative analysis of multi-omics datasets, including 3D genomics, epigenomics, and transcriptomics data. TAD boundaries are enriched with diverse architectural proteins, among which we paid more attention to CTCF-regulated ones. CTCF serves as a boundary insulator in addition to serving as a transcription factor (TF), and after the loss of CTCF from TAD boundaries, long-distance DNA–DNA interactions that extend beyond the confines of individual TADs occur [15,32]. Hence, CTCF-enriched boundaries play multifaceted roles and require further research. Moreover, two distinct mechanisms of TAD reorganization, namely, inter-TAD and intra-TAD, play pivotal roles in governing gene transcription (**Figure 1**A and B). Intra-TAD reorganization is the more commonly recognized mechanism occurring within TADs and may activate or inactivate gene transcription merely by the dynamic tuning of chromosomal looping [33–35] (Figure 1A). However, inter-TAD is usually based on TAD reorganization, and gene promoters and regulatory CREs may be separated by TAD boundaries or connected via the formation of chromosomal loops in the same TAD [36] (Figure 1B). These two mechanisms were evaluated via our Inter3D analysis for the identification of target oncogenes and tumor suppressor genes from the perspective of intra-TAD (mechanism 1, CTCF promotes the promoter–CRE interaction) or inter-TAD (mechanism 2, CTCF impedes the promoter–CRE interaction) (Figure S1A and B). Specifically, we focused on the candidate promoter–enhancer interactions regulated by CTCF-mediated interactome alterations in a series of experiments. In detail, Inter3D first identified CTCF-mediated TAD boundary alterations using high-throughput chromosome conformation capture (Hi-C) and CTCF chromatin immunoprecipitation sequencing (ChIP-seq) datasets (Figure 1C, steps 1 and 2). Next, incorporating the RNA sequencing (RNA-seq) datasets, Inter3D identified the candidate DEGs between variant samples regulated by CTCF binding alteration and TAD alteration (Figure 1C, step 3). Finally, Inter3D identified the candidate enhancers with robust promoter-distal peaks in assay for transposase-accessible chromatin with high-throughput sequencing (ATAC-seq) datasets, ruling out false- positive target genes regulated by enhancers only rather than by 3D structures (Figure 1C, step 4).

Next, to test our Inter3D method, we first constructed multiscale genome-wide chromatin contact maps with *in situ* Hi-C (Table S1) in both normal (ARPE19; Figure 1D, Figure S2A) and tumor (WERI-RB1; Figure 1E, Figure S2B) cells. We examined the A and B compartments, which represent active and inactive regions, respectively, by aggregating 100-kb bins of contiguous compartments. The A and B compartments of the two cell lines were widely varied (Figure S2C and D). Statistically, both ARPE19 and WERI-RB1 cells had similar numbers of A and B compartments; however, ARPE19 cells exhibited an opposite trend, *i.e.*, a slightly greater length distribution of the A compartment than that of the B compartment, compared to the pattern observed in WERI-RB1 cells (Figure 1F and G; Table S2). The majority of A/B compartments were preserved, with genome-wide compartment scores showing a moderate correlation (*r* = 0.4) between WERI-RB1 and ARPE19 cells (Figure 1H). Compartment A exhibited a slightly higher GC content than compartment B in WERI-RB1, while the GC content seemed to be the same in ARPE19 (Figure S2E and F). Overall, these analyses reveal the genomic architecture differences between normal and tumor cells and indicate the potential diversity of more subtle structures.

### The Inter3D pipeline

The candidate CRE–gene interaction can be identified by our CTCF-mediated Inter3D algorithm via the following steps. First, Inter3D identified tumor-specific CTCF-mediated TAD boundary alterations by identifying overlapping differential Hi-C TAD boundaries and CTCF ChIP-seq regions. The differential TAD boundaries that co-occurred with the differential CTCF-binding sites were selected for further analysis. Second, Inter3D was used to identify the top DEGs and promoters located around selected CTCF-binding sites as well as differential TAD boundaries, and genes with consistent promoter accessibility were filtered. The selected genes and promoters might be regulated by CTCF-binding alterations and TAD alterations. Finally, Inter3D identified candidate peak–gene pairs satisfying the criteria shown in Figure 1A and B. Specifically, for mechanism 1 (intra-TAD, loop alteration), the novel CTCF-mediated TAD boundary would insulate the regulation between the enhancer and gene, and the loss of such a TAD boundary would recover the regulome. The candidate peaks should (1) be located proximally upstream (< 20 kb) of the candidate gene and alter the CTCF-mediated TAD boundary, (2) be accessible to chromatin sites in both cases, and (3) involve novel CTCF resulting in gene deactivation or CTCF loss leading to gene activation. For mechanism 2 (inter-TAD, TAD reorganization), the novel CTCF-mediated TAD boundary enhanced the regulation between the enhancer and gene, and the loss of such a TAD boundary inactivated the regulome. The candidate peaks should (1) be located upstream (< 100 kb) of the candidate gene and have an altered CTCF-mediated TAD boundary, (2) be accessible at a chromatin site in both cases, and (3) involve novel CTCF resulting in gene activation or CTCF loss leading to gene deactivation.

### Dataset preprocessing

#### Hi-C library preparation

A total of 1 × 10^7^ cells were fixed with 1% formaldehyde for 10 min at room temperature, and the reaction was quenched with glycine. The fixed cells were permeabilized in 1 ml of lysis buffer [10 mM Tris-HCl pH 8.0, 10 mM NaCl, 0.2% NP-40, and Protease Inhibitor Cocktail (Catalog No. 200-664-3, Sigma, Darmstadt, Germany)] on ice for 15 min, followed by centrifugation at 2500 *g* for 5 min at 4°C. The resulting pellet was resuspended in 345 μl of 1× NEBuffer r2.1 (Catalog No. B6002V, New England Biolabs, Beverley, MA) and incubated with 38 μl of 1% sodium dodecyl sulfate (SDS) at 65°C for 10 min. Subsequently, 43 μl of 10% Triton X-100 (Catalog No.9036-19-5, Sigma) was added to quench the SDS at 37°C for 15 min. For chromatin digestion, 12 μl of 10× NEBuffer r2.1 and 400 U of *Hin*dIII (Catalog No. R3104V, New England Biolabs) were added per aliquot, and the mixture was gently mixed and incubated overnight at 37°C on a rocking platform. The *Hind*III restriction enzyme was then inactivated at 65°C for 25 min. Next, Biotin-14-dATP (Catalog No. 19524016, Life Technologies, Waltham, MA), dCTP, dGTP, dTTP, and DNA Polymerase I Klenow (Catalog No. M0210V, New England Biolabs) were added and incubated at 37°C for 2 h. The digested chromatin was diluted and religated using T4 DNA Ligase (Catalog No. M0202, New England Biolabs), followed by incubation at 16°C for 4 h with manual shaking performed three times. The chromatin products were decrosslinked overnight by adding 50 μl of 10 mg/ml Proteinase K (Catalog No. 39450-01-6, Sigma) and incubating at 65°C overnight. DNA was extracted using a phenol–chloroform extraction protocol. To remove RNA, 1 μl of RNase A (Catalog No. RNASEA-RO, Sigma) was added and incubated at 37°C for 30 min. T4 DNA Polymerase (Catalog No. M0203, New England Biolabs) was then used to remove biotin from unligated ends for 4 h at 20°C, with subsequent inactivation of the enzymes for 20 min at 75°C. DNA was purified and sheared to a size of 350 bp Covaris (Catalog No. M220, Covaris, Woburn, MA). DNA size selection was carried out using AMPure XP Beads (Catalog No. A63882, Beckman Coulter, Burea, CA). Biotin-ligated products were isolated with MyOne Streptavidin C1 Beads (Catalog No. 65001, Life Technologies). Subsequent steps included end repair, A-addition, an adaptor addition reaction, polymerase chain reaction (PCR) amplification, and DNA product size selection. The resulting libraries were sequenced using the Illumina PE150 paired-end sequencing platform. Reagent or resource used in this study are listed in Table S3.

#### ATAC-seq library preparation

Omni-ATAC, an enhanced ATAC-seq protocol, was used [37]. A total of 50,000 cells were lysed in 1 ml of cold ATAC-seq resuspension buffer (RSB; 10 mM Tris-HCl pH 7.4, 10 mM NaCl, and 3 mM MgCl_2_) for 3 min and then centrifuged at 500 *g* for 5 min at 4°C. The resulting cell pellets were resuspended in 50 μl of ATAC-seq RSB containing 0.1% NP-40, 0.1% Tween-20, and 0.01% digitonin, followed by incubation on ice for 3 min. After lysis, 1 ml of ATAC-seq RSB containing 0.1% Tween-20 (without NP-40 or digitonin) was added and thoroughly mixed. Nuclei were centrifuged at 500 *g* for 10 min at 4°C. The nuclei were then resuspended in 50 μl of transposition mix, consisting of 25 μl of 2× TD buffer (20 mM Tris-HCl pH 7.6, 10 mM MgCl_2_, and 20% dimethyl formamide), 2.5 μl of transposase (100 nM final concentration), 16.5 μl of phosphate-buffered saline (PBS), 0.5 μl of 1% digitonin, 0.5 μl of 10% Tween-20, and 5 μl of sterile H_2_O. Transposition reactions were incubated at 37°C for 30 min in a thermomixer with shaking at 1000 r/min. Transposed DNA was purified using the MinElute PCR Purification Kit (Catalog No. 28004, Qiagen, Dusseldorf, Germany) and eluted in 10 μl of EB buffer. Subsequent steps included end repair, A-addition, an adaptor addition reaction, and PCR amplification, which were performed sequentially. The resulting libraries were sequenced using the Illumina PE150 paired-end sequencing platform.

#### RNA extraction and library preparation

Total RNA was extracted from ARPE19 and WERI-RB1 cells using TRIzol Reagent (Catalog No. 15596026CN, Invitrogen, Waltham, MA). The purity and concentration of RNA were confirmed using a 2100 Bioanalyzer (Catalog No. G2939BA, Agilent Technologies, Santa Clara, CA) and a Qubit 2.0 Fluorometer (Catalog No. Q32866, Invitrogen) with a Qubit RNA Assay Kit (Catalog No. Q32852, Life Technologies). For RNA sample preparation, 2 μg of total RNA per sample was used as input material. Library preparation was performed using the VAHTS Total RNA-seq Library Prep Kit for Illumina (Catalog No. NR605-01/02, Vazyme, Nanjing, China) following the manufacturer’s recommendations. The resulting libraries were subjected to sequencing using the Illumina PE150 paired-end sequencing platform.

#### ATAC-seq data analysis

Briefly, with the help of Bowtie2 [38], we first mapped the ATAC-seq data generated in this study to the reference genome hg19 with default parameters. Subsequently, we removed multiple aligned reads and PCR duplications using SAMtools [39]. To further remove the impact of problematic regions in the human genome, we employed the Encyclopedia of DNA Elements (ENCODE) blacklist [40] and filtered out reads aligned to these regions using BEDTools [41]. Furthermore, we excluded reads mapped to the mitochondrial chromosome. Finally, peaks were called using MACS2 [42] with a *q* value cutoff of 0.05, incorporating a control. Replicate analysis of ATAC-seq replicates was conducted using ENCODE irreproducible discovery rate (IDR) tools [43] to enhance reliability.

#### RNA-seq data analysis

We initiated the process by eliminating low-quality reads and trimming adapter sequences using Trim Galore for read alignment and expression quantification. Subsequently, the remaining paired-end reads were aligned to the reference genome hg19 using STAR [44]. Unique mapped reads were counted using htseq-count [45], and the read count was normalized using the trimmed mean of M values (TMM) and transformed into reads per kilobase per million mapped reads (RPKM) through edgeR [46]. To ensure robustness, genes with an expression below an RPKM of 1 in at least one cell line sample were filtered out as low-abundance genes. DEGs were identified using edgeR [46], which considered genes with an overall false discovery rate (FDR) less than 0.05 and a fold change (FC) above 2.0.

#### Hi-C data analysis

To identify valid Hi-C interactions, we aligned these interactions to the reference genome hg19 using HiC-Pro [47]. We eliminated duplicate reads, assigned reads to *Hin*dIII restriction fragments, and filtered out invalid interactions by using HiC-Pro. The interactions were then binned at a resolution of 100 kb for compartmentation analysis and 40 kb for TAD identification. For compartment analysis, we initially smoothed the 100 kb matrix using the smoothMat function implemented in HiCRep [48] at the bin level. Subsequently, we computed the dominant eigenvectors of the smoothed contact matrices through the matrix2compartment.pl script from https://github.com/dekkerlab/cworld-dekker and smoothed compartmentation at the TAD level. TADs were classified into either A or B compartments based on the average dominant eigenvector of each TAD.

#### Public data collection

CTCF-binding sites of WERI-RB1 were collected from the CTCF ChIP-seq ENCODE data portal (ENCODE: ENCFF924OMX) [49]. The CTCF-binding sites of ARPE19 were collected from the Gene Expression Omnibus database (GEO: GSE60024) [49,50]. The ARPE19 CTCF-binding sites were converted from the hg18 genome to the hg19 genome using the University of California Santa Cruz (UCSC) Genome Browser Database LiftOver tool. The H3K27ac and H3K4me3 ChIP-seq data were collected from the ENCODE data portal (ENCODE: ENCFF949IKU and ENCFF272MMD).

### Evaluation and application in association studies

#### Cell lines

The retinoblastoma cell lines WERI-RB1 and Y79 were purchased from American Type Culture Collection (ATCC) and cultured in Roswell Park Memorial Institute (RPMI) 1640 medium (Catalog No. 12633012, GIBCO, Waltham, MA). ARPE19 and HEK293T cells were cultured in DMEM (Catalog No. 12491015, GIBCO, Waltham, MA). All media were supplemented with 10% fetal bovine serum (FBS; Catalog No. A5669701, GIBCO) and 1% penicillin/streptomycin (Catalog No. 15140122, GIBCO). All cells were incubated at 37°C and 5% CO_2_.

#### Chromosomal conformation capture experiment assay

For chromatin cross-linking, 1 × 10^6^ cells were resuspended in lysis buffer [10 mM Tris-HCl pH 8.0, 10 mM NaCl, 0.2% NP-40, and Protease Inhibitor Cocktail (Catalog No. 200-664-3, Sigma)] and incubated on ice for 30 min. The removal of lysis buffer was performed by centrifugation at 2500 *g* for 10 min at 4°C. The pellet was resuspended in 10 μl of 1× rCutSmart Buffer (Catalog No. B6004V, New England Biolabs) and incubated with 3 μl of 10% SDS at 37°C for 1 h. Subsequently, 9 μl of 10% Triton X-100 (Catalog No. 9036-19-5, Sigma) was added to quench the SDS at 37°C for 1 h. Furthermore, 100 μl of 1× rCutSmart Buffer (Catalog No. B6004V, New England Biolabs) and 600 U of *Hae*III (Catalog No. R0108V, New England Biolabs) were added to different tubes and mixed gently to digest the chromatin overnight at 37°C on a rocking platform. The next day, the restriction enzyme was inactivated at 65°C for 20 min. The DNA was resuspended in 1× ligation buffer [30 mM Tris-HCl pH 8.0, 10 mM MgCl_2_, 10 mM DL-dithiothreitol (DTT; Catalog No. 3483-12-3, Sigma), and 1 mM adenosine triphosphate (ATP; Catalog No. 11140965001, Sigma)] along with T4 DNA Ligase (Catalog No. M0202, New England Biolabs) and incubated at 16°C for 4 h. After incubation at room temperature for 30 min without shaking, the chromatin products were decrosslinked overnight by adding 30 μl of 10 mg/ml Proteinase K (Catalog No. 39450-01-6, Sigma) and incubated at 65°C overnight. Phenol/chloroform was used to extract DNA. PCR and Sanger sequencing were carried out to confirm long-range chromatin interactions, and the PCR primers used are listed in Table S4.

#### Luciferase assay

Cells were harvested, and genomic DNA (gDNA) was extracted using a Genomic DNA Extraction Kit (Catalog No. DP304, TIANGEN, Beijing, China) following the manufacturer’s instructions. Subsequently, the gDNA was utilized for PCR amplification with Q5 High-Fidelity DNA Polymerase (Catalog No. M0491, New England Biolabs). The primers used for PCR are listed in Table S4. Fragments corresponding to potential enhancers, specifically myosin light chain 12B (*MYL12B*) E1 and cytochrome P450 family 27 subfamily B member 1 (*CYP27B1*) E2, were cloned and inserted into the pGL3-Promoter Vector (Catalog No. E1751, Promega, Madison, WI). HEK293T cells in a 24-well plate were allowed to reach 50% confluence within 24 h before transfection. A cotransfection approach was employed by introducing 900 ng of pGL3-E1/E2-promoter or pGL3-promoter and 90 ng of pRL-TK (Catalog No. E2241, Promega) into HEK293T cells using Lipofectamine 3000 (Catalog No. 18324012, Invitrogen). After 48 h, luciferase activity was assessed using the Dual Luciferase Reporter Gene Assay Kit (Catalog No. 11402ES60, YEASEN, Shanghai, China) according to the manufacturer’s instructions. Each experimental group was replicated with three technical repeats, and the transfections were performed in three independent experiments. Firefly luciferase activity was normalized to Renilla luciferase activity, and *P* values were calculated using Student’s *t*-test.

#### RNA extraction and real-time quantitative polymerase chain reaction

Total RNA was extracted from cells utilizing TRIzol Reagent (Catalog No. 15596026CN, Invitrogen), and complementary DNA (cDNA) was synthesized using 1st Strand cDNA Synthesis SuperMix (Catalog No. 11123ES60, YEASEN). Real-time quantitative polymerase chain reaction (RT-qPCR) was conducted with qPCR SYBR Green Master Mix (Catalog No. 11198ES08, YEASEN) on a Roche LightCycler 96 system (Catalog No. 05015219001, Roche, Basel, Switzerland). The presented data illustrated the FC of the experimental group compared to that of the control group. Briefly, △Ct was calculated as △Ct = Ct (test gene) − Ct (reference gene). The ΔΔCt was calculated as ΔΔCt = ΔCt (experimental group) − ΔCt (control group). The FC of a test gene in the experimental group *versus* the control group was computed as FC = 2^−ΔΔCt^. Each gene was tested in triplicate in every independent experiment, and all experiments were performed in triplicate. The primers used are listed in Table S4.

#### Western blot

Cells were lysed in Radio-Immunoprecipitation Assay (RIPA) Lysis Buffer (Catalog No. 20101ES60, YEASEN) containing 1 mM phenylmethanesulfonyl fluoride (PMSF; Catalog No. 20104ES03, YEASEN) for 30 min and then centrifuged at 13,000 *g* for 10 min at 4°C. Protein samples were separated by sodium dodecyl sulfate–polyacrylamide gel electrophoresis (SDS–PAGE) in 12% (w/v) polyacrylamide gels and transferred to polyvinylidene fluoride membranes (Catalog No. GVWP02500, Millipore, Boston, MA). After blocking with 5% bovine serum albumin (BSA; Catalog No. 36101ES25, YEASEN) for 1 h at room temperature, the membrane was incubated with different antibodies in 5% BSA overnight at 4°C. Subsequently, the membrane was incubated with Peroxidase-Conjugated Goat Anti-Rabbit IgG (Catalog No. 33118ES60, YEASEN). The band signals were visualized and quantified using the fully automatic chemiluminescence/fluorescence image analysis system (Catalog No. 5200, Tanon, Shanghai, China). The following antibodies were used in this study: anti-MYL12B (1:1000; Catalog No. ab137063, Abcam, Cambridge, UK), anti-CYP27B1 (1:750; Catalog No. A1716, ABclonal, Wuhan, China), and anti-ACTB (1:5000; Catalog No. ab119716, Abcam).

## Results

### Alterations in the TAD boundary are the predominant features of the tumor genome

**Figure 2:**
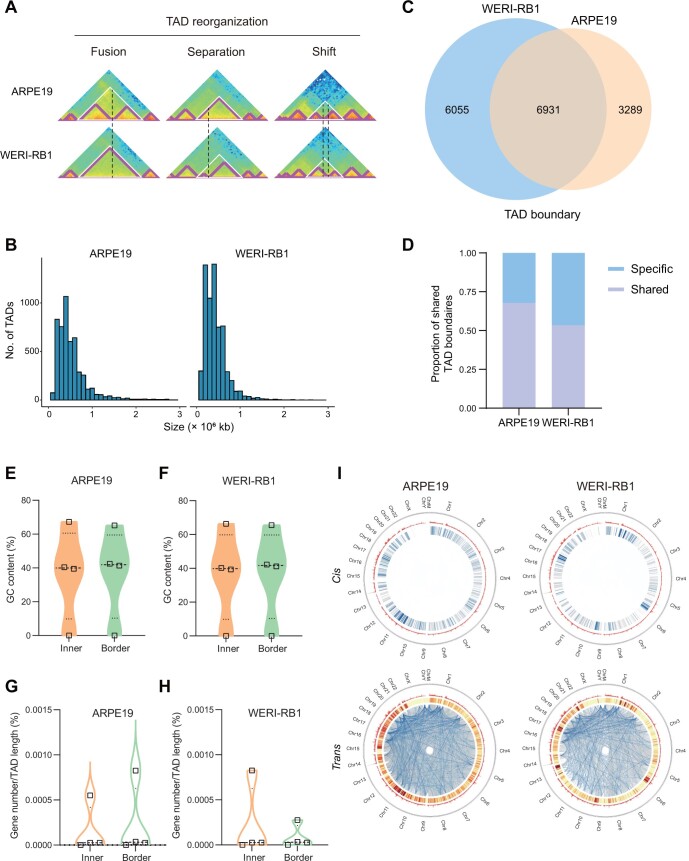
TAD rearrangement in retinoblastoma cells **A**. TAD reorganization types of chromosome 21 in ARPE19 (top) and WERI-RB1 (bottom) cells at 40-kb resolution. **B**. Number of TADs of different sizes in ARPE19 (left) and WERI-RB1 (right) cells. **C**. Venn diagram showing the overlap of TAD boundaries between ARPE19 and WERI-RB1 cells. **D**. Proportion of shared TAD boundaries in ARPE19 and WERI-RB1 cells. **E**. and **F**. Violin and box plots showing the GC content of the TAD inner and border of ARPE19 (E) and WERI-RB1 (F) cells. **G**. and **H**. Violin and box plots showing the gene density of the TAD inner and border of ARPE19 (G) and WERI-RB1 (H) cells. **I**. Circos plots of genome-wide significant *cis*-interaction and *trans*-interaction sites in ARPE19 and WERI-RB1 cells at 10-kb resolution. For *cis*-interaction sites, from outward to inward are chromosome distribution, gene density (red), and *cis*-interaction site links (ReadCount value from large to small; blue from dark to light). For *trans*-interaction sites, from outward to inward are chromosome distribution, gene density (red), and *trans*-interaction distribution (number from small to large; from yellow to red).

To further uncover what larger compartments were composed of and why they varied between normal and tumor cells, we analyzed the TAD structure by calculating the directionality index (DI) value for each bin using the method described by Dixon and colleagues [11]. This analysis included TAD position information and a structure diagram of WERI-RB1 and ARPE19 with 24 chromosomes at 40-kb resolution. Different TAD alteration types, such as fusion, separation, and shift, occurred frequently when we compared TADs of WERI-RB1 cells to those of ARPE19 cells, taking chromosome 21 as an example (**Figure 2**A). We identified a total of 5110 and 6493 TADs for ARPE19 and WERI-RB1 cells, respectively, and most TADs were smaller than 1 × 10^6^ kb (Figure 2B; Table S5). According to the results of the TAD analysis, the bin where the start and end of the calculated TAD are located can be regarded as the separation zone, or the TAD boundary, of the two TADs. It was found that 6931 TAD boundaries are shared between ARPE19 and WERI-RB1 cells (Figure 2C). The shared TAD boundaries accounted for nearly 68% and 53% of all boundaries, respectively, in ARPE19 and WERI-RB1 cells (Figure 2D). The inner and border of TADs exhibited similar GC content in WERI-RB1 and ARPE19 cells (Figure 2E and F), while the gene density of the TAD inner and border showed significant differences. Contrary to the findings in ARPE19 cells (Figure 2G), the gene density of the TAD border was significantly lower than that of the TAD inner in WERI-RB1 cells (Figure 2H). In most mammalian cells, including normal ARPE19 cells, gene density is typically higher at TAD borders. However, our investigation revealed an intriguing departure from this norm. Specifically, in tumor WERI-RB1 cells, we noted a contrasting pattern in which gene density was heightened at TAD inners (Figure 2H). Aberrant DNA replication characterizes tumor cells, and cell immortalization during cancer development leads to uncontrolled cell proliferation. The replication origin repertoire was disturbed after cell immortalization in immortalized cell lines obtained by the misexpression of oncogenes, and immortalization through oncogenic gene expression resulted in decreased origin density at TAD borders [51]. In our study, a wild-type tumor retinoblastoma cell line, WERI-RB1, was used to generate Hi-C data. This finding may indicate that complex heterogeneity and gene transcription disorders could lead to aberrant gene density at the TAD inner and border in tumor cells.

We further identified significant *cis*-interactions and *trans*-interactions using the Fit-Hi-C [52] and Xie et al. [53] methods (Table S6). After sorting genome-wide significant interaction sites of WERI-RB1 and APRE19 from large to small by the ReadCount value (the number of reads that support the interaction; ReadCount > 2; *P* ≤ 0.01; *q* ≤ 0.01) at 10-kb resolution, the first 25,000 pairs were displayed as links on the Circos graph (Figure 2I). A comparison of the significant interactions detected between ARPE19 and WERI-RB1 cells revealed 55,129 pairs and 25,470 pairs, respectively, of significant *cis*-interaction and *trans*-interaction sites repeated between the two groups (Figure S3A). However, the number of significant interaction sites specific to individual cells was several times greater than that shared among cells. This observation strongly suggests the potential significance of interaction variations in the process of tumorigenesis (Figure S3A). We next analyzed the enrichment of DEGs in the specific TADs in WERI-RB1 *versus* ARPE19 (Figure S3B) and conducted Gene Ontology (GO) classification and Kyoto Encyclopedia of Genes and Genomes (KEGG) enrichment analyses, and found that the DEGs were related to nervous and sensory systems and signal transduction (Figure S3C and D). All these data indicate that our Hi-C data comprehensively sketch the multiscale architecture of the tumor genome and increase our interest in effectively capturing significant interaction changes caused by TAD boundary alterations.

### Inter3D identifies TAD reorganization-mediated gene loci within the 3D epigenetic regulatory landscape

**Figure 3:**
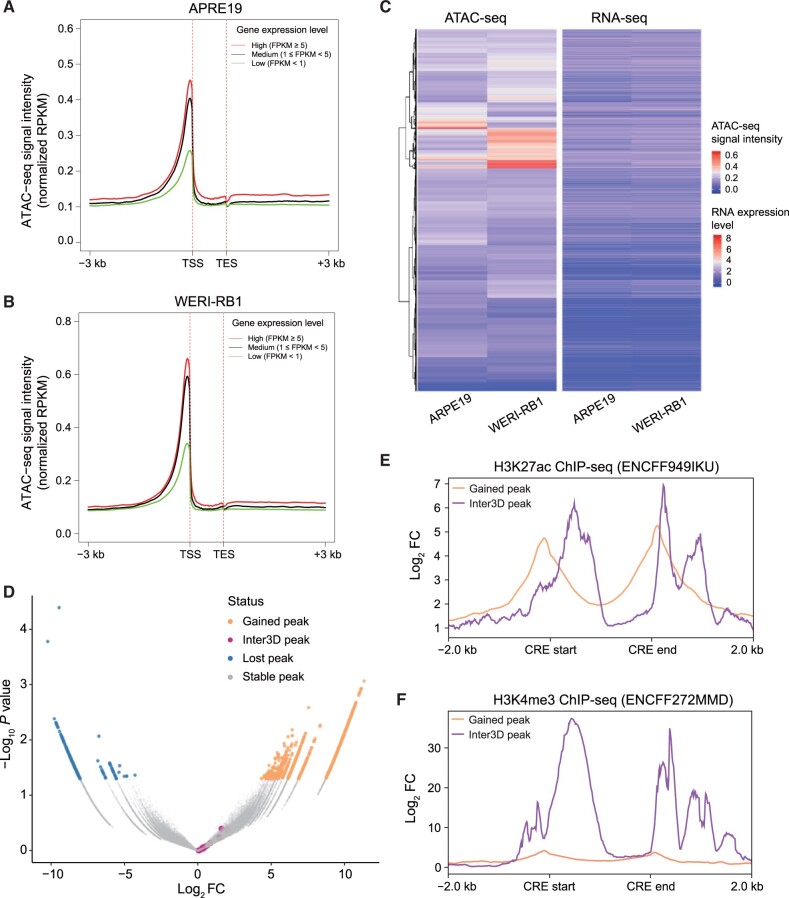
Characterization of a 3D epigenetic regulatory map conjugating TAD reorganization **A**. and **B**. Relationship between ATAC-seq signal intensity and gene expression level in ARPE19 (A) and WERI-RB1 (B) cells. **C**. Clustering of ATAC-seq signal intensity and gene expression in ARPE19 and WERI-RB1 cells. **D**. Volcano plot of DARs in ARPE19 cells *versus* WERI-RB1 cells. Orange dots indicate DAR peaks gained by WERI-RB1 cells compared to ARPE19 cells; blue dots indicate DAR peaks lost by WERI-RB1 cells compared to ARPE19 cells; gray dots indicate DAR peaks shared by WERI-RB1 and ARPE19 cells; purple dots indicate Inter3D-identifed shared DAR peaks between WERI-RB1 and ARPE19 cells. **E**. and **F**. Aggregation plot of H3K27ac (E) and H3K4me3 (F) ChIP-seq signals on differentially gained peaks (orange line) and Inter3D peaks (purple line) in WERI-RB1 cells. DAR, differentially accessible region; RPKM, reads per kilobase per million mapped reads; TSS, transcription start site; TES, transcription end site; FC, fold change.

To further assess the biological significance of TAD reorganization with Inter3D, we also conducted RNA-seq for gene expression, ATAC-seq for chromatin accessibility, and CTCF ChIP-seq for CTCF occupancy. We first performed quality control analysis on ATAC-seq and RNA-seq datasets (Tables S7 and S8). The insert size distributions of the two ATAC-seq datasets were in line with expectations (Figure S4A and B). The ATAC-seq signals in WERI-RB1 and ARPE19 cells were significantly enriched near the transcription start sites (TSSs) (Figure S4C). Additionally, we evaluated the consistency of the two replicates using the IDR, which indicates the reliability of our data quality (Figure S4D–G). The total ATAC-seq peak numbers of ARPE19 and WERI-RB1 were 59,220 and 48,055, respectively, and their peak widths were similar (Figure S5A and B). The distribution of ATAC-seq peaks in both groups was dominated by promoter regions, introns, and distal genes (Figure S5C). ARPE19 and WERI-RB1 cells had fewer common differentially accessible regions (DARs) but showed large differences in chromatin accessibility (Figure S5D). Based on these findings, we further divided the gene expression levels into high, medium, and low categories (**Figure 3**A and B) and found that the expression levels of the genes were positively correlated with the ATAC-seq signal intensity near the TSSs (Figure 3C). We then conjointly analyzed the genes in DARs located in promoter regions and detected overlapping DEGs, TAD alteration-regulated genes, and CTCF-binding genes via conventional methods; unfortunately, these conventional methods could not identify enough candidate targets regulated by inter-TAD or intra-TAD mechanisms (Figure S5E). However, with the Inter3D method, we successfully identified 8 oncogenes and 28 suppressor genes based on intra-TAD (mechanism 1) and 11 oncogenes and 26 suppressor genes based on inter-TAD (mechanism 2) (Figure S1B).

To investigate the characteristics of the Inter3D candidates, we further compared them with differentially accessible CREs between ARPE19 and WERI-RB1 cells, as differential analysis is commonly applied to identify candidate functional CREs [54]. We found that Inter3D candidates had no overlap with condition-specific CREs (Figure 3D). We further evaluated the enhancer activity and promoter activity of these non-differential Inter3D candidates. Inter3D candidates showed similar enhancer potential (Figure 3E) and greater promoter activity (Figure 3F) than did WERI-RB1-specific peaks, indicating characteristics of both enhancer and promoter in Inter3D candidates. Overall, using our proposed Inter3D method, we identified variant TAD reorganization-mediated gene loci and active enhancer–promoter interactions in an automatic and comprehensive way.

### TAD separation interrupts the intrachromosomal loop and is correlated with decreased *MYL12B* transcription

**Figure 4:**
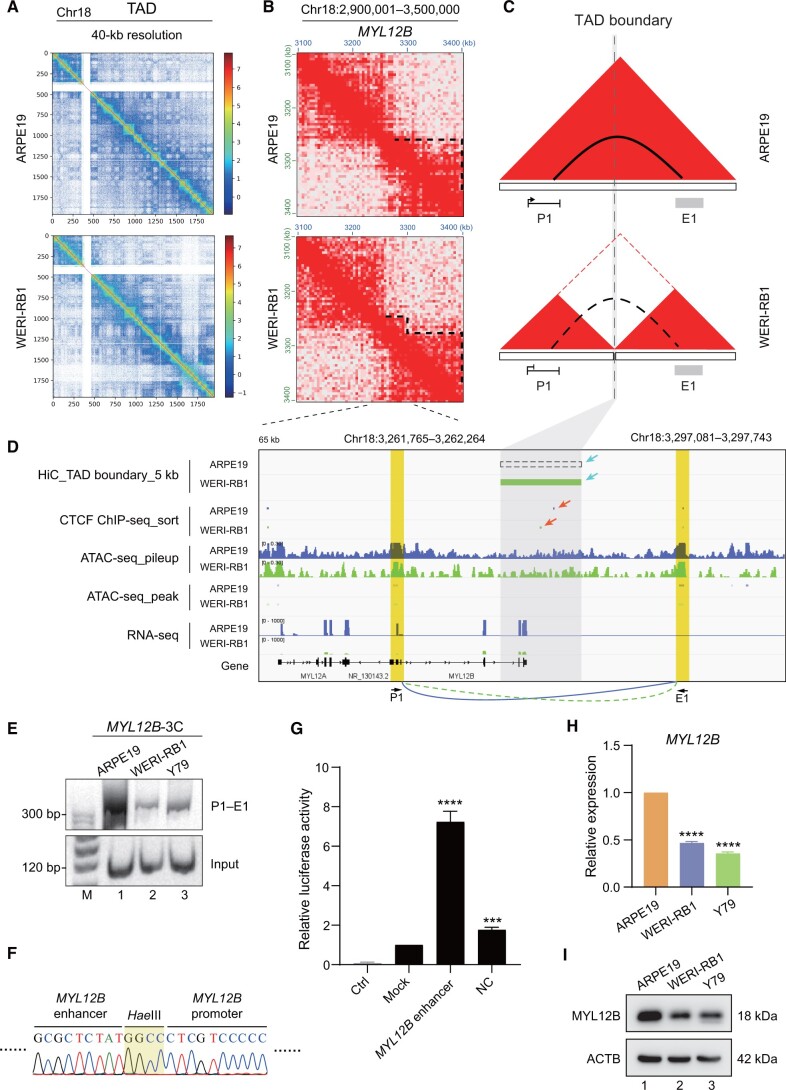
TAD separation hinders the formation of an intrachromosomal loop in the *MYL12B* locus **A**. TAD distribution of chromosome 18 in ARPE19 (top) and WERI-RB1 (bottom) cells at 40-kb resolution. **B**. Chromatin interaction heatmaps at 10-kb resolution at the *MYL12B* locus. Dotted lines represent existing TADs. **C**. Models depicting how TAD separation interrupts the formation of the promoter–enhancer chromosomal loop. Red triangles represent TADs; dotted line represents TAD boundary; solid arc represents intrachromosomal loop; and dotted arc represents nonexistent loop. **D**. TAD boundary visualization as well as CTCF ChIP-seq, ATAC-seq, and RNA-seq tracks for the *MYL12B* locus. Blue tracks represent sequencing data of ARPE19 cells, and green tracks represent sequencing data of WERI-RB1 cells. Dashed rectangle represents the nonexistent TAD boundary in ARPE19 cells, and gray rectangle represents the mimic neo-TAD insulation in WERI-RB1 cells analyzed from Hi-C data. The different TAD boundaries and CTCF peaks between the ARPE19 and WERI-RB1 cell lines are indicated with blue and red arrows, respectively. The gray area indicates the TAD boundary locus. The yellow areas indicate the promoter (P1, left) and enhancer (E1, right) of *MYL12B*. **E**. PCR of 3C experiments for detecting the chromosomal loop between the *MYL12B* promoter and the downstream potential enhancer sequence. The primer pair P1–E1 was used. **F**. DNA sequencing of the chromosomal loop amplified in (E). The grass-green area means the *Hae*III cutting site. **G**. RT-qPCR analysis of the luciferase activity of the downstream E1 DNA fragment of *MYL12B*. Ctrl indicates the control group without any firefly reporter plasmids; mock indicates the group with wild-type firefly reporter plasmids; *MYL12B* enhancer indicates the group with downstream E1 DNA fragment contained in firefly reporter plasmids; NC indicates the negative control group with a random fragment of equal length contained in firefly reporter plasmids. Renilla reporter plasmids were included in all the experimental groups. The data were calculated as the ratio of firefly to Renilla luciferase activity (Fluc/Rluc) in the dual luciferase reporter system. For comparison, the ratio of Fluc/Rluc in the mock group was arbitrarily set as 1 in the calculation. **H**. RT-qPCR analysis of *MYL12B* expression at the RNA level. **I**. Western blot analysis of *MYL12B* expression at the protein level. ***, *P* < 0.001; ****, *P* < 0.0001 (Student’s *t*-test). 3C, chromosomal conformation capture; PCR, polymerase chain reaction; RT-qPCR, real-time quantitative polymerase chain reaction; ACTB, beta-actin.

Considering the principle that TAD separation regulates promoter–enhancer chromosomal looping within gene loci, we selected *MYL12B*, which is one of the most prominently regulated genes in the cytoskeletal compartment [55], to further confirm the accuracy of the Inter3D method. As expected, TAD separation was observed in the *MYL12B* locus on chromosome 18 in WERI-RB1 cells *versus* in ARPE19 cells at 40-kb resolution (**Figure 4**A and B). Unlike in ARPE19 cells, there was a neo-TAD boundary as an interval resulting in TAD separation between the *MYL12B* promoter (P1) and its downstream potential enhancer (E1) in WERI-RB1 cells (Figure 4C). The formation of the neo-TAD boundary and TAD separation resulted in the specific loss of a chromosomal loop between the *MYL12B* P1 promoter and the E1 region, leading to a reduction in *MYL12B* transcription in WERI-RB1 cells (Figure 4D). To further confirm whether the above identified TAD separation can cause alterations in promoter–enhancer chromosomal looping, we performed chromosomal conformation capture (3C) experiments. The 3C results demonstrated that a novel chromosomal loop between the *MYL12B* P1 promoter and its distal potential E1 enhancer was present in control ARPE19 cells (Figure 4E, lane 1), and DNA sequencing confirmed the presence of this novel chromosomal loop (Figure 4F). However, this chromosomal looping was diminished after TAD separation in the WERI-RB1 and Y79 retinoblastoma cells (Figure 4E, lanes 2 and 3). A dual luciferase reporter assay confirmed that the E1 DNA fragment downstream of the *MYL12B* P1 promoter had enhancer activity (Figure 4G). To determine whether the loss of this chromosomal looping would change *MYL12B* transcription in retinoblastoma cells, we measured the expression of *MYL12B.* We found that the expression of *MYL12B* at both the RNA (Figure 4H) and protein (Figure 4I) levels was significantly lower in tumor cells than in normal ARPE19 cells. Taken together, these findings collectively illustrate that the Inter3D algorithm plays a pivotal role in discerning distinct interactions between gene promoters and CREs that are hindered by TAD separation. These interactions are subsequently correlated with the altered expression levels of genes, as exemplified by the case of *MYL12B*, which is facilitated through a chromosomal loop.

### TAD fusion generates chromosomal interactions and is potentially linked to *CYP27B1* activation

**Figure 5:**
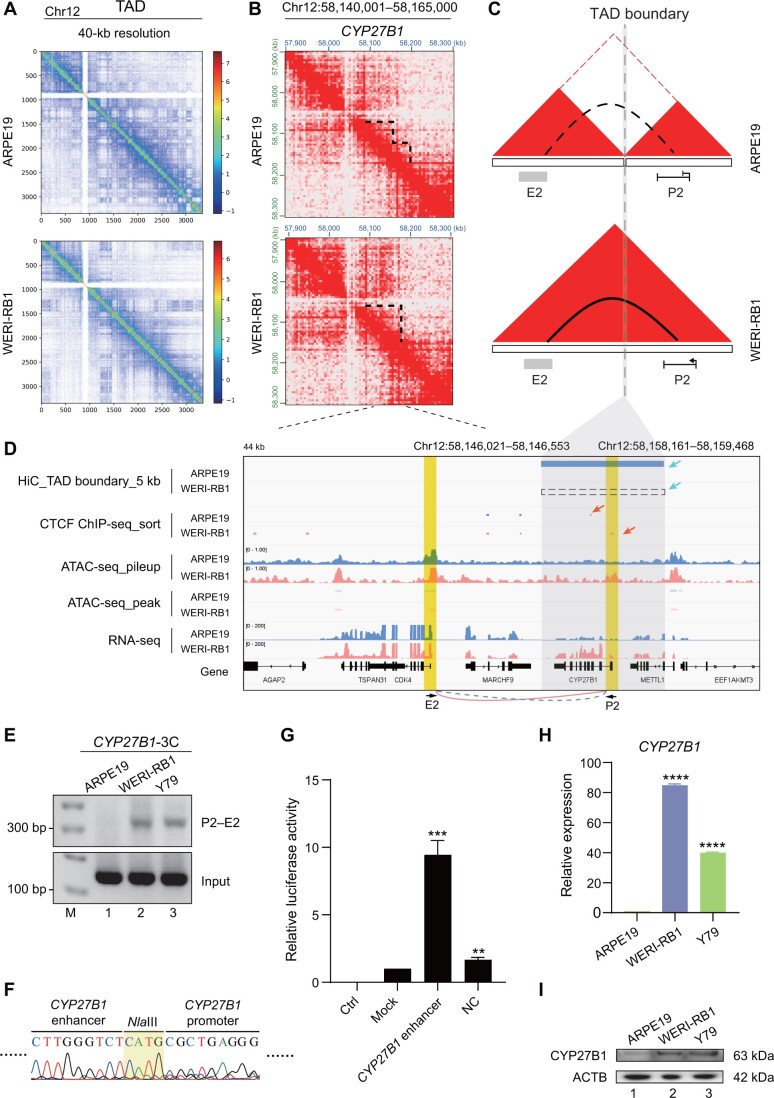
TAD fusion contributes to chromosomal looping in the *CYP27B1* locus **A**. TAD distribution of chromosome 12 in ARPE19 (top) and WERI-RB1 (bottom) cells at 40-kb resolution. **B**. Chromatin interaction heatmaps at 10-kb resolution at the *CYP27B1* locus. Dotted lines represent existing TADs. **C**. Models depicting how TAD fusion contributes to the formation of the promoter–enhancer chromosomal loop. Red triangles represent TADs; dotted line represents TAD boundary; solid arc represents intrachromosomal loop; and dotted arc represents nonexistent loop. **D**. TAD boundary visualization as well as CTCF ChIP-seq, ATAC-seq, and RNA-seq tracks for the *CYP27B1* locus. Blue tracks represent sequencing data of ARPE19 cells, and red tracks represent sequencing data of WERI-RB1 cells. Bule rectangle represents the mimic TAD insulation in ARPE19 cells analyzed from Hi-C data, and dotted rectangle represents the nonexistent TAS boundary in WERI-RB1 cells. The different TAD boundaries and CTCF peaks between the ARPE19 and WERI-RB1 cell lines are indicated with blue and red arrows, respectively. The gray area indicates the TAD boundary. The yellow areas indicate the promoter (P2, right) and enhancer (E2, left) of *CYP27B1*. **E**. PCR of 3C experiments for detecting the chromosomal loop between the *CYP27B1* promoter and the downstream potential enhancer sequence. The primer pair P2–E2 was used. **F**. DNA sequencing of the chromosomal loop amplified in (E). The grass-green area means the *Nla*III cutting site. **G**. RT-qPCR analysis of the luciferase activity of the downstream E2 DNA fragment of *CYP27B1*. Ctrl indicates the control group without any firefly reporter plasmids; mock indicates the group with wild-type firefly reporter plasmids; *CYP27B1* enhancer indicates the group with downstream E2 DNA fragment contained in firefly reporter plasmids; NC indicates the negative control group with a random fragment of equal length contained in firefly reporter plasmids. Renilla reporter plasmids were included in all the experimental groups. The data were calculated as the ratio of firefly to Renilla luciferase activity (Fluc/Rluc) in the dual luciferase reporter system. For comparison, the ratio of Fluc/Rluc in the mock group was arbitrarily set as 1 in the calculation. **H**. RT-qPCR analysis of *CYP27B1* expression at the RNA level. **I**. Western blot analysis of *CYP27B1* expression at the protein level. **, *P* < 0.01; ***, *P* < 0.001; ****, *P* < 0.0001 (Student’s *t*-test).

We next selected the *CYP27B1* locus, which is involved in granulomatous disease and tumors such as lymphomas [56], to construct a model of TAD fusion-mediated chromosomal looping and gene transcription activation. We found that TAD fusion of individual TADs occurred near the *CYP27B1* locus on chromosome 12 in WERI-RB1 cells *versus* in ARPE19 cells at 40-kb resolution (**Figure 5**A and B). In control ARPE19 cells, the *CYP27B1* promoter (P2) was located in one TAD, while its potential enhancer (E2) was located in another TAD (Figure 5C, upper). However, the *CYP27B1* P2 promoter and the potential E2 enhancer were located in the same TAD in WERI-RB1 cells (Figure 5C, bottom). Loss of the TAD boundary and the presence of a new TAD fusion resulted in the specific formation of a chromosomal loop between the *CYP27B1* P2 promoter and the E2 region, leading to an increase in *CYP27B1* transcription in WERI-RB1 cells (Figure 5D). To further confirm that our identified TAD fusion across the *CYP27B1* locus would change this promoter–enhancer chromosomal looping, a 3C assay demonstrated that a novel chromosomal loop between the *CYP27B1* P2 promoter and its distal potential E2 enhancer formed in both WERI-RB1 and Y79 tumor cells (Figure 5E, lanes 2–3) compared with that in ARPE19 cells (Figure 5E, lane 1). DNA sequencing further confirmed the presence of this novel chromosomal loop (Figure 5F). A luciferase assay proved that the potential E2 DNA fragment downstream of the *CYP27B1* P2 promoter had enhancer activity (Figure 5G). To determine whether chromosomal looping would change *CYP27B1* transcription, we examined the RNA and protein expression of *CYP27B1* in tumor cells and found that it was significantly higher than that in normal ARPE19 cells (Figure 5H and I). In conclusion, these findings collectively demonstrate the contribution of the Inter3D algorithm in revealing TAD fusion-induced interactions between gene promoters and CREs. These interactions are correlated with the up-regulated expression of genes such as *CYP27B1* mediated by chromosomal loops.

## Discussion

Recent studies have illuminated the significant role of TAD reorganization in orchestrating chromosome topology and governing gene expression dynamics [57]. TADs enriched with developmentally regulated chromatin exhibit heightened levels of evolutionary conservation and constrained gene expression patterns. These TADs might exert a more pronounced influence on gene regulation, potentially owing to an elevated frequency of long-range enhancer–promoter interactions associated with developmental genes [23]. However, methodologies for revealing the regulatory impact of TAD reorganization on gene transcription across diverse cancer types remain limited. A handful of investigations have reported TAD-mediated gene regulation at specific loci during tumorigenesis [58,59]. Others have concentrated on establishing frameworks for identifying CRE rearrangements, such as enhancer hijacking, which drives gene dysregulation in cancer contexts [60]. In this study, we introduced the innovative Inter3D algorithm, which enables the concurrent identification of genome-wide DEGs alongside alterations in TAD boundaries within the context of cancer. We successfully pinpointed two genomic loci, namely, *MYL12B* and *CYP27B1*, by leveraging our Inter3D algorithm-driven multi-omics analyses. Within these loci, we observed instances of TAD separation or fusion influencing the formation of chromosomal loops between individual promoters and enhancers. This finding supports an alternative mechanism underlying TAD formation and highlights its potential regulatory role in governing gene expression. Notably, Inter3D provides an efficient tool for the preselection of potential genes modulated through TAD reorganization.

Some studies have shown that TAD boundary alterations play important roles in chromosome topology and gene expression [36,61]. In contrast, other studies have seemingly observed modest effects on gene transcriptional changes caused by TAD alterations [62,63], which raises controversy about the functional importance of TADs for gene regulation. Epigenetic modifications and the inherent heterogeneity of cells play pivotal roles in cell fate determination [64–66]. The intricate orchestration of gene expression is subject to a diverse array of genetic and epigenetic influences. Although TADs might emerge as predominant regulators of gene transcription in certain instances, it is essential to recognize that the broader regulatory landscape entails a multitude of components. These components include factors such as RNA splicing, TFs, histone modifications, R-loop structures, and RNA–DNA triplexes, each of which is capable of exerting pronounced effects on gene expression modulation [64,65,67]. The involvement of TADs in this intricate regulatory network might not be a stand-alone determinant of gene expression outcomes. Instead, TADs could synergistically collaborate with these multifaceted factors, contingent upon the specific genomic locus in question. Further exploration is warranted to elucidate the precise dynamics of these collaborations and their implications for gene expression control. This holistic perspective highlights the contextual nature of gene regulation and underscores the need for comprehensive investigations to elucidate the nuanced interplay of these regulatory elements.

The lack of a comprehensive method to identify these TAD alteration-mediated functional gene loci was considered to be one of the causes of this controversy. Generally, active promoters or enhancers can be identified by ChIP-seq or ATAC-seq by following the principle of consistent peak tendency with RNA-seq. Recently, Cicero [68] was developed to predict connections between distal CREs and target gene promoters with co-accessibility for single-cell ATAC-seq, which helped elucidate the genome-wide *cis*-regulatory network. Gene promoters and regulatory CREs are usually separated by TAD boundaries and located in different TADs or connected by the formation of chromosomal loops within the same TAD [29]. However, robust tools for the capture of these CRE–promoter contacts within or between TADs are lacking. In this study, for the first time, we developed a novel Inter3D algorithm to identify TAD alteration-mediated functional genes by integrating complex multi-omics information, which may be an example of the importance of the universal TAD reorganization mechanism underlying gene transcription. Previous CRE regulation prediction algorithms usually lack CTCF and comprehensive multi-omics data [9,63]. In contrast, Inter3D incorporates CTCF ChIP-seq, RNA-seq, ATAC-seq, and Hi-C data, which significantly reduces the candidate pool for CRE–gene identification, making it less suitable for mathematical or machine learning modeling as well. Nevertheless, direct comparative analysis of multi-omics data in Inter3D offers general applications for identifying CTCF-regulated CRE interactions. This inherent limitation restricts the capacity of the algorithm for comprehensive evaluation across a broader spectrum of real-world samples and extensive data cohorts. The potential of Inter3D remains promising, and its wider application and performance assessment within diverse biological contexts and expansive datasets have yet to be fully explored.

The interaction of MYL12B with MAFG-AS1 promotes the proliferation and migration of hepatocellular carcinoma cells [69], and *CYP27B1* is a risk gene for malignant tumors and other chronic diseases [56,70,71]. The regulatory mechanism underlying *MYL12B* transcription remains understudied. The transcriptional control of *CYP27B1* can be hormonally modulated at the DNA level through methylation/demethylation switching, where active DNA demethylation leads to transcriptional repression [72]. Additionally, the *CYP27B1* promoter can be bound by two TF families, nuclear factor-kB (NF-KB) and CCAAT/enhancer-binding protein beta (CEBPB), which stimulate its transcription [73]. Remarkably, our study uniquely and comprehensively delineated the potential relationship between TAD alterations and the expression of both *MYL12B* and *CYP27B1* for the first time. This study provides a novel perspective on the transcriptional regulation of these genes. The potential influence of other chromatin regulatory elements on the transcriptional regulation of *MYL12B* and *CYP27B1*, whether in a positive or negative manner, cannot be discredited in theory. Consequently, further exploration to better comprehend the intricate regulation of these genes is of paramount importance. Therefore, it is of considerable interest to ascertain whether TAD reorganization can indeed reshape the expression of target genes in the context of tumorigenesis, as substantiated by experimental evidence. Additionally, delving deeper into the relationship between gene transcription and TAD alterations, as identified through our Inter3D method, holds promise for future research.

Gene expression can be regulated by different mechanisms at multiple levels, including *cis*-acting elements and *trans*-acting factors, from pretranscription to post-translation modifications. TAD reorganization encompasses three primary classifications: TAD separation, fusion, and shift, all of which are orchestrated by alterations in TAD boundaries [9]. Within our Hi-C contact map analysis, we discerned the presence of all three types. However, our primary focus was on TAD fusion and separation, primarily due to their pronounced alterations in boundaries. Notably, the TAD shift category remains an intriguing avenue for our future research endeavors. The disruption of TADs leads to ectopic DNA–DNA interactions between CREs and promoters, driving the dysregulation of genes that leads to altered cell fate and causes diseases [9,74–78]. However, the regulatory mechanism underlying TAD-mediated gene expression remains unclear. Since several gene transcription events are triggered by TAD reorganization in many studies, it is possible that those genes may be regulated by a similar TAD pattern, as shown in our study, thereby providing alternative avenues for the exploration of TAD alteration-guided gene expression. Therefore, whether our TAD reorganization patterns function universally in variant genes or specifically in our case is of potential interest and needs to be further explored.

## Code availability

The Inter3D pipeline used in this study is freely available at the GitHub (https://github.com/bm2-lab/inter3D), Zenodo (https://zenodo.org/record/7857813#.ZEYooexByjB), figshare (https://figshare.com/projects/inter3D_ucsc_genome_tracks/165706), and BioCode (https://ngdc.cncb.ac.cn/biocode/tool/BT7399).

## Supplementary Material

qzae034_Supplementary_Data

## Data Availability

The raw sequence data of Hi-C, ATAC-seq, and RNA-seq in this study have been deposited in the NCBI GEO database (GEO: GSE230798), which are publicly accessible at https://www.ncbi.nlm.nih.gov/geo/, and have also been deposited in the Genome Sequence Archive for Human [79] at the National Genomics Data Center, Beijing Institute of Genomics, Chinese Academy of Sciences / China National Center for Bioinformation (GSA-Human: HRA007411), which are publicly accessible at https://ngdc.cncb.ac.cn/gsa-human/.

## CRediT author statement

**Tianyi Ding:** Conceptualization, Investigation, Methodology, Visualization, Writing – original draft, Writing – review & editing. **Shaliu Fu:** Conceptualization, Software, Methodology, Visualization, Writing – original draft, Writing – review & editing. **Xiaoyu Zhang:** Investigation, Data curation, Visualization. **Fan Yang:** Investigation, Validation. **Jixing Zhang:** Methodology, Investigation. **Haowen Xu:** Resources. **Jiaqi Yang:** Methodology. **Chaoqun Chen:** Data curation. **Yibing Shi:** Investigation. **Yiran Bai:** Investigation. **Wannian Li:** Data curation. **Xindi Chang:** Validation. **Shanjin Wang:** Supervision. **Chao Zhang:** Supervision. **Qi Liu:** Conceptualization, Supervision. **He Zhang:** Project administration, Funding acquisition, Conceptualization, Supervision, Writing – original draft. All authors have read and approved the final manuscript.

## ORCID

0000-0001-9092-0262 (Tianyi Ding)

0000-0003-1707-5474 (Shaliu Fu)

0000-0003-0242-8118 (Xiaoyu Zhang)

0009-0002-5444-1621 (Fan Yang)

0009-0007-5520-4229 (Jixing Zhang)

0009-0002-2303-5532 (Haowen Xu)

0009-0000-6331-9148 (Jiaqi Yang)

0009-0009-3762-1840 (Chaoqun Chen)

0009-0005-1549-0553 (Yibing Shi)

0009-0007-5606-7270 (Yiran Bai)

0009-0004-6232-6345 (Wannian Li)

0009-0000-2804-6463 (Xindi Chang)

0000-0001-7287-3642 (Shanjin Wang)

0000-0001-6418-8370 (Chao Zhang)

0000-0003-2578-1221 (Qi Liu)

0000-0001-9954-4451 (He Zhang)
