# Supplementary material for: Inter3D: Capture of TAD Reorganization Endows Variant Patterns of Gene Transcription
Source: Genomics Proteomics Bioinformatics. 2024 May 8;22(3):qzae034. doi: 10.1093/gpbjnl/qzae034 (PMC12016567; doi:10.1093/gpbjnl/qzae034)
Supplement: qzae034_Supplementary_Data [file qzae034_supplementary_data.zip › Supplementary Table 3-done.docx]

Table S3 Reagent or resource used in this study

| **Reagent or re****source** | **Source** | **Identifier** |
| --- | --- | --- |
| **Antibodies** | | |
| Myosin light chain 12B (MYL12B) | Abcam | Cat# ab137063 |
| Cytochrome P450 family 27 subfamily B member 1 (CYP27B1) | ABclonal | Cat# A1716 |
| Actin beta (ACTB) | Abcam | Cat# ab119716 |
| Goat Anti-Rabbit IgG | YEASEN | Cat# 33101ES60 |
| **Chemicals, peptides, and recombinant proteins** | | |
| Dulbecco''s Modified Eagle Medium (DMEM) medium | Invitrogen | Cat# C11965500CP |
| Roswell Park Memorial Institute (RPMI)-1640 medium | Invitrogen | Cat# C11875500CP |
| Fetal bovine serum (FBS) | Invitrogen | Cat# C11875500CP |
| Penicillin Streptomycin Solution | Invitrogen | Cat# 15140122 |
| Dulbecco phosphate-buffered saline (DPBS) | Invitrogen | Cat# C14190500CP |
| Trypsin 0.25% ethylene diamine tetraacetic acid (EDTA) | Invitrogen | Cat# 25200072 |
| Opti minimal essential medium (MEM) I | Invitrogen | Cat# 11058021 |
| Lipofectamine 3000 | Invitrogen | Cat# L3000015 |
| Puromycin | Invitrogen | Cat# A1113802 |
| HindIII | NEB | Cat# R3104 |
| NlaIII | NEB | Cat# R0125 |
| HaeIII | NEB | Cat# R0108 |
| AluI | NEB | Cat# R0137 |
| SalI | NEB | Cat# R3138 |
| BamHI | NEB | Cat# R3136 |
| DNA Polymerase I Klenow fragment | NEB | Cat# M0212 |
| T4 DNA Ligase | NEB | Cat# M0202 |
| Q5 High-Fidelity DNA Polymerase | NEB | Cat# M0493 |
| Biotin-14-dATP | Thermo Fisher | Cat# 19518018 |
| Proteinase K | YEASEN | Cat# 10401ES60 |
| Glycogen | Thermo Fisher | Cat# R0561 |
| **Critical commercial assays** | | |
| TRIzol Regent | Sigma | Cat# T9424 |
| EndoFree Plasmid Midi Kit | Omega | Cat# D6915-03 |
| Gel Extraction Kit | Omega | Cat# D2500-02 |
| Genomic DNA Kit | TIANGEN | Cat# DP304 |
| AMPure XP Beads | Beckman Coulter | Cat# A63880 |
| Dynabeads MyOne Streptavidin C1 Beads | Thermo Fisher | Cat# 65002 |
| QUBIT ASSAY TUBES, SET OF 500 | Thermo Fisher | Cat# Q32856 |
| QUBIT DSDNA HS ASSAY KIT, 500 | Thermo Fisher | Cat# Q32854 |
| **Experimental models: cell lines** | | |
| HEK293T cell | Cell Bank of SIBCB | Cat# SCSP-502 |
| ARPE19 cell | Cell Bank of SIBCB | Cat# GNHu45 |
| WERI-RB1 cell | Cell Bank of SIBCB | Cat# TCHu213 |
| Y79 cell | ATCC | Cat# HTB-18 |
| **Software and algorithms** | | |
| Inter3D | This study |  |
| **Other** | | |
| Polyvinylidene fluoride (PVDF) membrane | Millipore |  |
| Covaris Sonicator M220 | Covaris |  |
| Agilent 2100 Bioanalyzer | Agilent |  |
