# Supplementary material for: Inter3D: Capture of TAD Reorganization Endows Variant Patterns of Gene Transcription
Source: Genomics Proteomics Bioinformatics. 2024 May 8;22(3):qzae034. doi: 10.1093/gpbjnl/qzae034 (PMC12016567; doi:10.1093/gpbjnl/qzae034)
Supplement: qzae034_Supplementary_Data [file qzae034_supplementary_data.zip › Supplementary Table 7-done.docx]

Table S7 Basic statistics and quality control for ATAC-seq data

| **Type** | **ARPE19** | **WERI-RB1** |
| --- | --- | --- |
| Raw read pairs | 50,842,500 | 32,300,389 |
| Clean read pairs | 48,962,192 | 31,446,416 |
| Duplicates rate | 31.26% | 37.99% |
| Valid read pairs | 29,613,124 | 14,947,926 |

*Note*: ATAC-seq, assay for transposase-accessible chromatin with high throughput sequencing.
