# Supplementary material for: Inter3D: Capture of TAD Reorganization Endows Variant Patterns of Gene Transcription
Source: Genomics Proteomics Bioinformatics. 2024 May 8;22(3):qzae034. doi: 10.1093/gpbjnl/qzae034 (PMC12016567; doi:10.1093/gpbjnl/qzae034)
Supplement: qzae034_Supplementary_Data [file qzae034_supplementary_data.zip › Supplementary Table 1-done.docx]

Table S1 Basic statistics and quality control for Hi-C data

| **Type** | **ARPE19** | |  | **WERI-RB1** | |
| --- | --- | --- | --- | --- | --- |
|  | **Num** | **%** |  | **Num** | **%** |
| Raw data reads | 2,023,558,540 |  |  | 2,011,001,760 |  |
| Raw data size | 303,533,781,000 |  |  | 301,650,264,000 |  |
| Clean data reads | 1,994,909,738 |  |  | 1,981,747,026 |  |
| Clean data size | 291,227,459,790 |  |  | 289,133,913,425 |  |
| Mapped side1* | 804,081,344 |  |  | 801,288,778 |  |
| Mapped side2* | 778,773,978 |  |  | 777,475,676 |  |
| Total DS reads (paired) | 670,968,214 | 100% |  | 669,427,589 | 100% |
| Same fragment reads removed | 106,489,134 |  |  | 109,811,780 |  |
| Self-circles | 338,990 |  |  | 205,115 |  |
| Dandling ends | 106,086,816 |  |  | 109,545,364 |  |
| Error | 63,328 |  |  | 61,301 |  |
| Extra dandling ends removed | 135,433,006 |  |  | 142,112,146 |  |
| Valid pairs | 429,046,074 | 63.94% |  | 417,503,663 | 62.37% |
| Duplicates removed | 33,822,136 |  |  | 58,567,912 |  |
| Reads after filtering | 395,223,938 | 58.90% |  | 358,935,751 | 53.62% |
| *Cis*-reads | 309,462,903 |  |  | 281,737,133 |  |
| *Trans*-reads | 85,761,035 |  |  | 77,198,618 |  |

*Note*: * Marked data of SE (single-end), the remaining data are from PE (paired-end). Hi-C, high-throughput chromosome conformation capture.
