# Supplementary material for: Inter3D: Capture of TAD Reorganization Endows Variant Patterns of Gene Transcription
Source: Genomics Proteomics Bioinformatics. 2024 May 8;22(3):qzae034. doi: 10.1093/gpbjnl/qzae034 (PMC12016567; doi:10.1093/gpbjnl/qzae034)
Supplement: qzae034_Supplementary_Data [file qzae034_supplementary_data.zip › Supplementary Table 5-done.docx]

Table S5 Numbers and length of TADs

| **Samples** | **Numbers of TADs** |  | **Length of TADs** | | | |
| --- | --- | --- | --- | --- | --- | --- |
|  |  |  | **Min** | **Median** | **Mean** | **Max** |
| ARPE19 | 5110 |  | 80,000 | 440,000 | 538,455 | 7,360,000 |
| WERI-RB1 | 6493 |  | 80,000 | 400,000 | 434,285 | 33,040,000 |

*Note*: TADs, topologically associating domains.
