# Supplementary material for: Inter3D: Capture of TAD Reorganization Endows Variant Patterns of Gene Transcription
Source: Genomics Proteomics Bioinformatics. 2024 May 8;22(3):qzae034. doi: 10.1093/gpbjnl/qzae034 (PMC12016567; doi:10.1093/gpbjnl/qzae034)
Supplement: qzae034_Supplementary_Data [file qzae034_supplementary_data.zip › Supplementary material captions.docx]

**Supplementary material**

**Supplementary figures**

**Figure S1 Schematic overview of the study**

**A.** Scheme of the workflow used in this study showing the integrative analysis and selection principles. The results were validated with experiments. **B.** The workflow of Inter3D applied to screen targets under four conditions: (1) *in situ* Hi-C for TAD alteration; (2) CTCF ChIP-seq for CTCF binding alteration; (3) RNA-seq for the top DEGs; and (4) ATAC-seq for different CREs. * indicates genes studied in this study.

**Figure S2 Differences in whole-genome chromatin interactions**

**A****.** and **B.** Genome-wide chromatin interaction heatmaps of all chromosomes in ARPE19 (A) and WERI-RB1 (B) cells at 500-kb resolution. **C.** and **D.** A/B compartment distribution and interaction heatmap of ARPE19 (C) and WERI-RB1 (D) cells on Chr18 at 100-kb resolution. Based on the interaction matrix of 100-kb resolution, a positive or negative C-score determined each 100-kb bin as the A (blue) or B (red) compartment, respectively. **E.** and **F.** The GC content of A/B compartments in ARPE19 (E) and WERI-RB1 (F) cells.

**Figure S3 Characterization of DEGs in TADs**

**A.** Numbers of genome-wide significant *cis*-interaction and *trans*-interaction sites in ARPE19 and WERI-RB1 cells. **B.** Total enrichment of DEGs in different TADs between ARPE19 and WERI-RB1. **C.** GO classification of DEGs in different TADs between ARPE19 and WERI-RB1. **D.** KEGG enrichment of DEGs in different TADs between ARPE19 and WERI-RB1. A means ARPE19 and W means WERI-RB1. GO, Gene Ontology; KEGG, Kyoto Encyclopedia of Genes and Genomes.

**Figure S4 Quality control of ATAC-seq data**

**A.** and **B.** Distribution of insert sizes in ARPE19 (A) and WERI-RB1 (B) cells. **C.** ATAC-seq signal enrichment around the TSS in ARPE19 and WERI-RB1 cells. **D****.** and **E.** Peak overlap between ARPE19 (D) and WERI-RB1 (E) samples and pseudo replicates (random data sampling). **F****.** and **G.** Evaluation of ARPE19 (F) and WERI-RB1 (G) sample data repeatability. IDR, irreproducible discovery rate.

**Figure S5 Analysis of gene distribution with multi-omics datasets**

**A.** and **B.** Total peak number and peak width of ATAC-seq in ARPE19 (A) and WERI-RB1 (B) cells. **C.** Distribution of ATAC-seq peaks aligned to the genome in ARPE19 (top) and WERI-RB1 (bottom). **D.** Peak overlap of ATAC-seq in ARPE19 and WERI-RB1 cells. Gray circle means WERI-RB1 and purple circle means ARPE19. **E.** Venn diagram showing the overlap among genes in DARs, DEGs, TAD alteration-regulated genes, and CTCF-binding genes.

**Table S1 Basic statistics and quality control for Hi-C data**

**Table S2 Number and length of A and B compartments**

**Table S3 Reagent or resource used in this study**

**Table S4 Primers used in this study**

**Table S5 Numbers and length of TADs**

**Table S6 Statistical results for significant *cis*-interactions and *trans*-interactions at 40-kb resolution**

**Table S7 Basic statistics and quality control for ATAC-seq data**

**Table S8 Basic statistics and quality control for RNA-seq data**
