# Supplementary material for: Inter3D: Capture of TAD Reorganization Endows Variant Patterns of Gene Transcription
Source: Genomics Proteomics Bioinformatics. 2024 May 8;22(3):qzae034. doi: 10.1093/gpbjnl/qzae034 (PMC12016567; doi:10.1093/gpbjnl/qzae034)
Supplement: qzae034_Supplementary_Data [file qzae034_supplementary_data.zip › Supplementary Table 4-done.docx]

Table S4 Primers used in this study

| **Primer name** | **Sequences (5'-3')** | **Purpose** |
| --- | --- | --- |
| GAPDH-F | TCCAAAATCAAGTGGGGCGA | RT-qPCR |
| GAPDH-R | TGATGACCCTTTTGGCTCCC | RT-qPCR（115） |
| MYL12B-F | TGATGAAGAAGCAACAGGCACC | RT-qPCR |
| MYL12B -R | GGATGCGTGTGAACTCGATGTA | RT-qPCR (119) |
| CYP27B1-F | AAGTTCGGACTGGAAGGCATC | RT-qPCR |
| CYP27B1-R | CAGCGTGGACACAAACACCGA | RT-qPCR (126) |
| MYL12Bp12-InputF | GCCGCCCTCACCTTCTCAACATTAC | 3C |
| MYL12Bp12-InputR | GAACCCTGAGTTGCCAGATGTAGC | 3C (113) |
| MYL12B-P1 | GAAGGGTGGCGGAGCTACCAAAG | 3C |
| MYL12B-P1N | GAAGGGTGGCGGAGCTACCAAAGGAGC | 3C |
| MYL12B-E1 | TCTAACGAGCTGCTGCAAGCGGAAC | 3C |
| MYL12B-E1N | TCTAACGAGCTGCTGCAAGCGGAACCCT | 3C |
| CYP27B1p-InputF | TTCAGGGTGCTCGCGAAAGAAAG | 3C |
| CYP27B1p-InputR | GTGCTAATCCCCAGCACAGACCAC | 3C (143) |
| CYP27B1-P2 | CGCGATGGAACACTCTGGAGGCGT | 3C |
| CYP27B1-E2 | AATCCTTCAGCCGGGAGTTGGGCT | 3C |
| DLRA-MYL12B-F | CGCGGATCCTCTAACGAGCTGCTG | Luciferase assay |
| DLRA-MYL12B-R | ACGCGTCGACATAGAGCGCCAAAAAATTTC | Luciferase assay |
| DLRA-CYP27B1-F | TATTGGATCCAGCTCGGTCCGGAGCAG | Luciferase assay |
| DLRA-CYP27B1-R | ACGCGTCGACGTAGTGAGACTGGAGTTC | Luciferase assay |

*Note*: 3C, chromosomal conformation capture; RT-qPCR, real-time quantitative polymerase chain reaction.
