# Supplementary material for: Inter3D: Capture of TAD Reorganization Endows Variant Patterns of Gene Transcription
Source: Genomics Proteomics Bioinformatics. 2024 May 8;22(3):qzae034. doi: 10.1093/gpbjnl/qzae034 (PMC12016567; doi:10.1093/gpbjnl/qzae034)
Supplement: qzae034_Supplementary_Data [file qzae034_supplementary_data.zip › Supplementary Table 6-done.docx]

Table S6 Statistical results for significant *cis*-interactions and *trans*-interactions at 40-kb resolution

|  | **Significant *cis*-interaction** | **Significant *trans*-interaction** |
| --- | --- | --- |
| ARPE19 | 277,212 | 3,470,298 |
| WERI-RB1 | 211,583 | 5,460,578 |
