# Supplementary material for: Inter3D: Capture of TAD Reorganization Endows Variant Patterns of Gene Transcription
Source: Genomics Proteomics Bioinformatics. 2024 May 8;22(3):qzae034. doi: 10.1093/gpbjnl/qzae034 (PMC12016567; doi:10.1093/gpbjnl/qzae034)
Supplement: qzae034_Supplementary_Data [file qzae034_supplementary_data.zip › Supplementary Table 8-done.docx]

Table S8 Basic statistics and quality control for RNA-seq data

| **Type** | **ARPE19** | **WERI-RB1** |
| --- | --- | --- |
| Clean reads pairs | 25,533,036 | 18,016,085 |
| Clean base (bp) | 7,659,910,800 | 5,404,825,500 |
| Length | 150; 150 | 150; 150 |
| Q20(%) | 97.7; 97.7 | 97.4; 97.5 |
| Q30(%) | 94.2; 93.9 | 93.6; 93.6 |
| GC(%) | 54.0; 53.9 | 53.4; 53.3 |

*Note*: RNA-seq, RNA sequencing.
