# Supplementary material for: Inter3D: Capture of TAD Reorganization Endows Variant Patterns of Gene Transcription
Source: Genomics Proteomics Bioinformatics. 2024 May 8;22(3):qzae034. doi: 10.1093/gpbjnl/qzae034 (PMC12016567; doi:10.1093/gpbjnl/qzae034)
Supplement: qzae034_Supplementary_Data [file qzae034_supplementary_data.zip › Supplementary Table 2-done.docx]

Table S2 Number and length of A and B compartments

| **Compartments** | | **Number of compartments** |  | **Length of compartments** | | | |
| --- | --- | --- | --- | --- | --- | --- | --- |
|  |  |  |  | **Min** | **Median** | **Mean** | **Max** |
| ARPE19 | A | 1,265 |  | 38,663 | 500,000 | 955,467 | 9,700,000 |
|  | B | 1,289 |  | 100,000 | 800,000 | 1,213,535 | 9,800,000 |
| WERI-RB1 | A | 1,209 |  | 100,000 | 500,000 | 962,344 | 17,700,000 |
|  | B | 1,254 |  | 38,663 | 700,000 | 1,274,353 | 18,300,000 |
