# Supplementary figures and images for: Inter3D: Capture of TAD Reorganization Endows Variant Patterns of Gene Transcription
Source: Genomics Proteomics Bioinformatics. 2024 May 8;22(3):qzae034. doi: 10.1093/gpbjnl/qzae034 (PMC12016567; doi:10.1093/gpbjnl/qzae034)

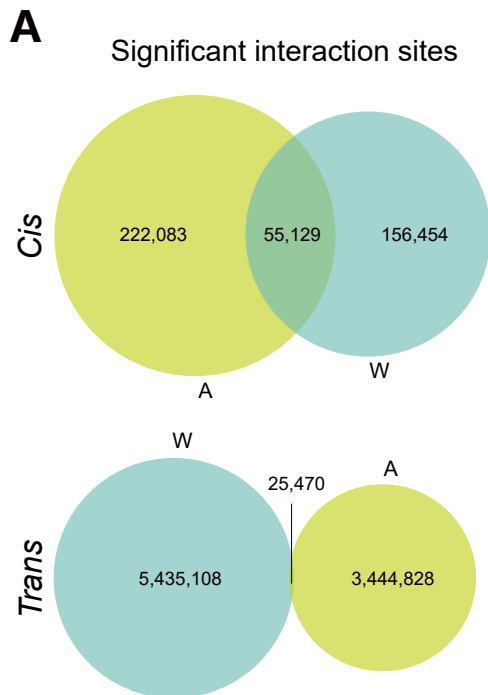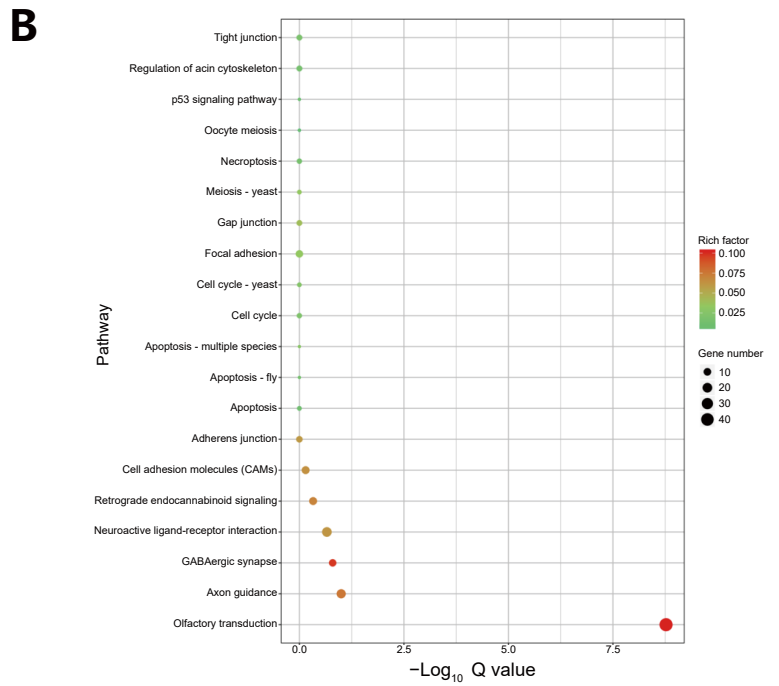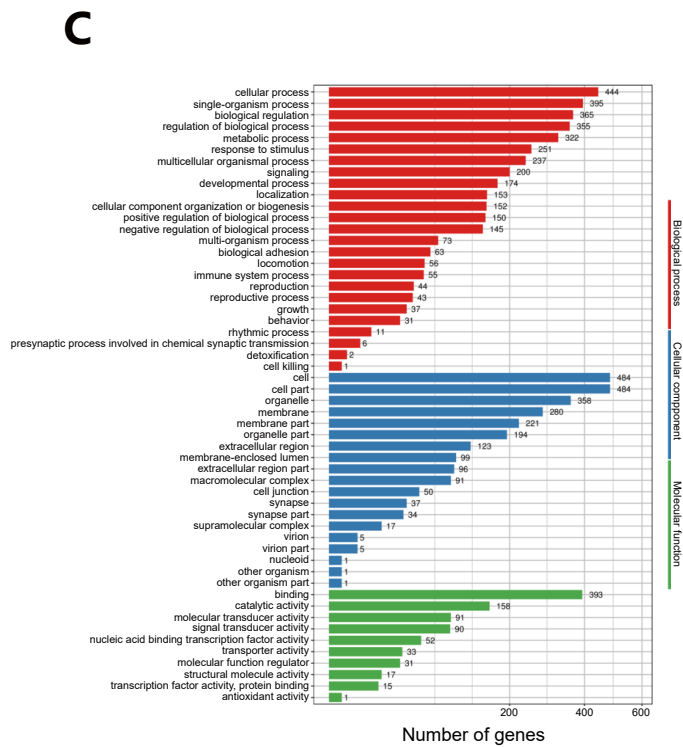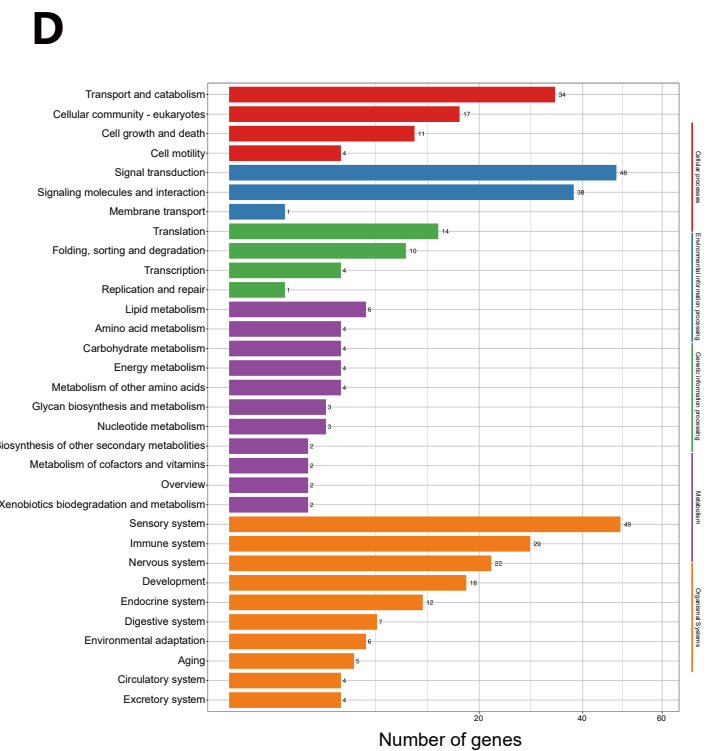

Supplement: qzae034_Supplementary_Data [file qzae034_supplementary_data.zip › Supplementary_Figure 3 E.pdf]

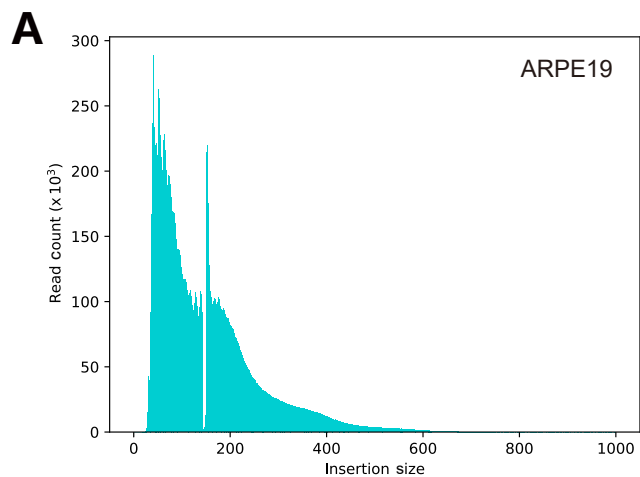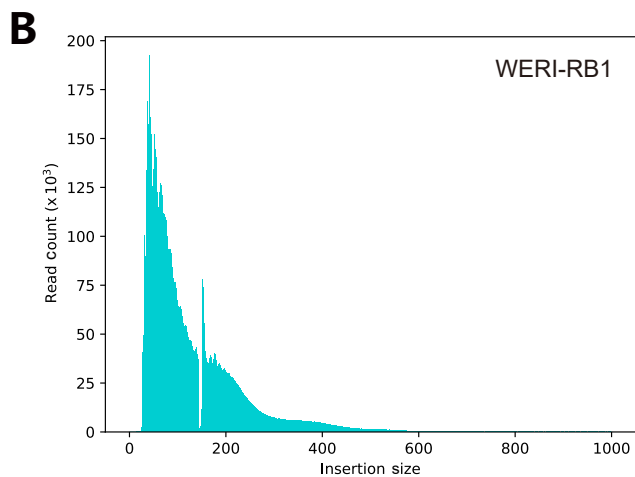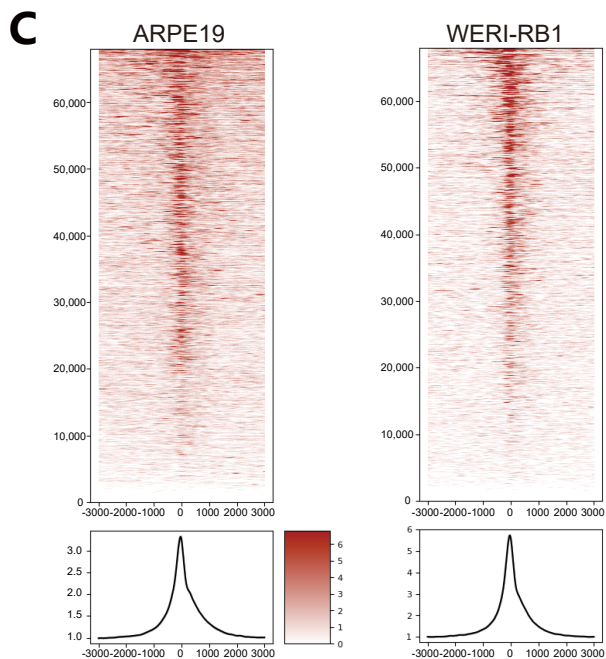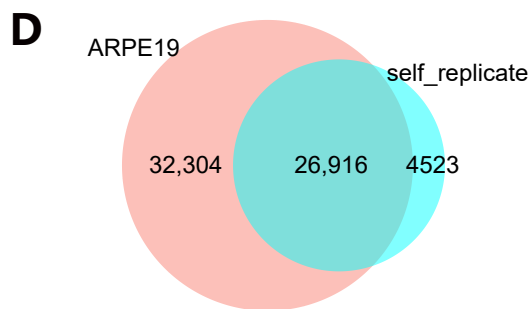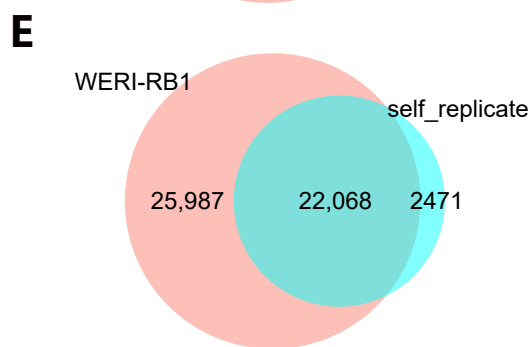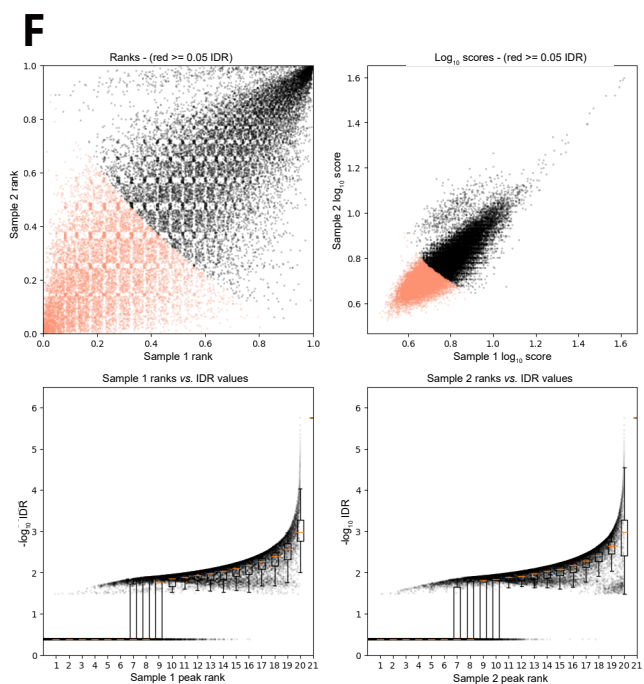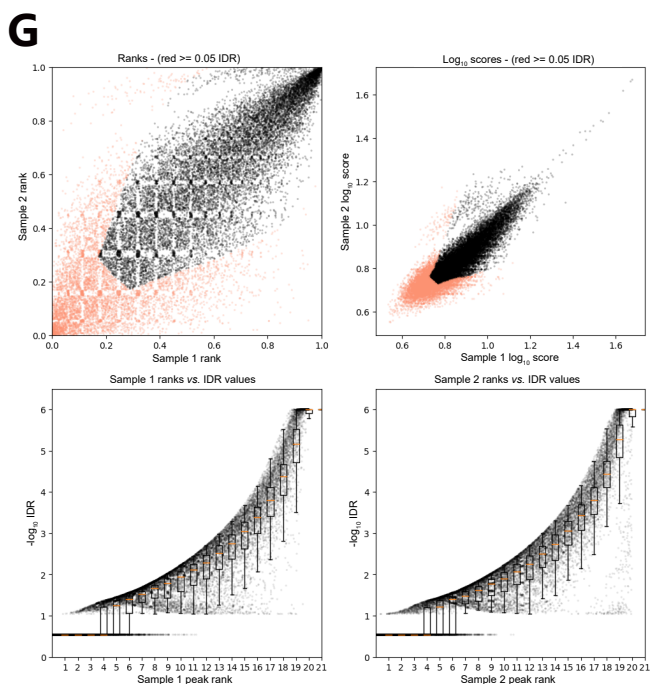

Supplement: qzae034_Supplementary_Data [file qzae034_supplementary_data.zip › Supplementary_Figure 4.pdf]

**A**

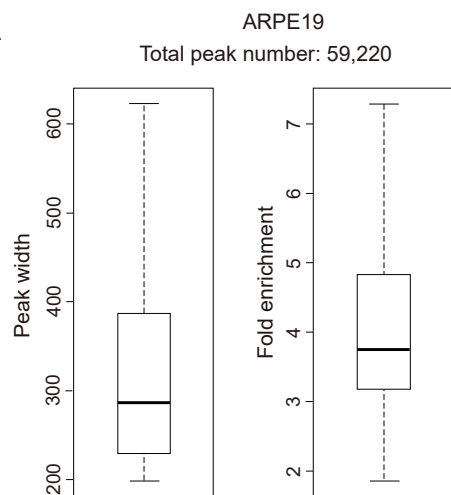

**B**

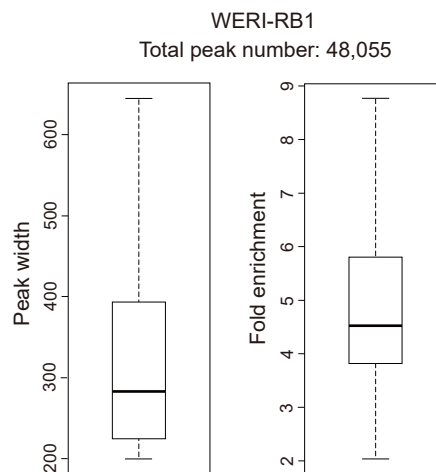

**C**

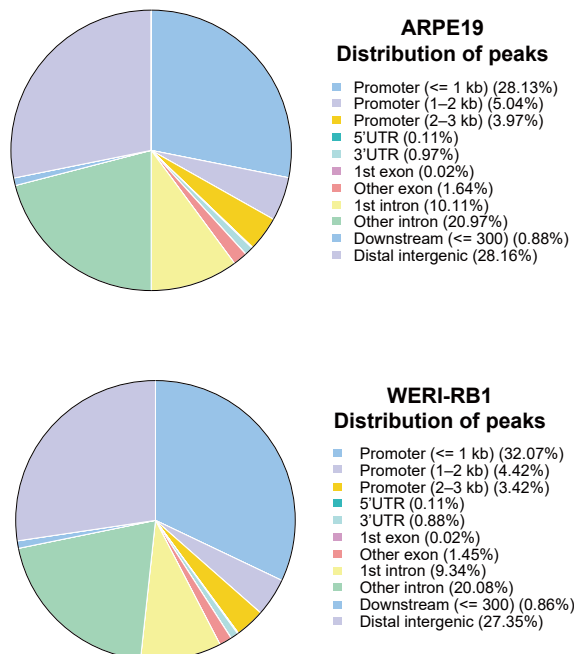

**D**

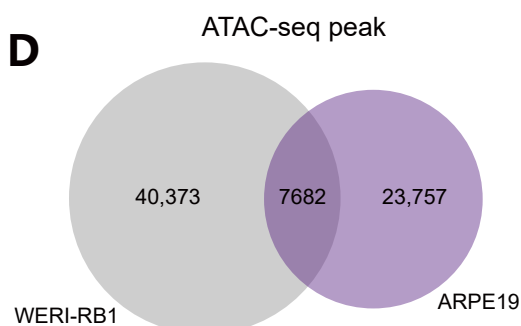

**E**

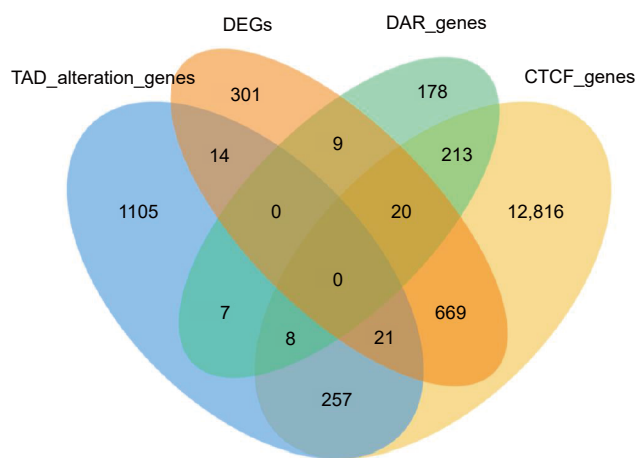

Supplement: qzae034_Supplementary_Data [file qzae034_supplementary_data.zip › Supplementary_Figure 5.pdf]

**A**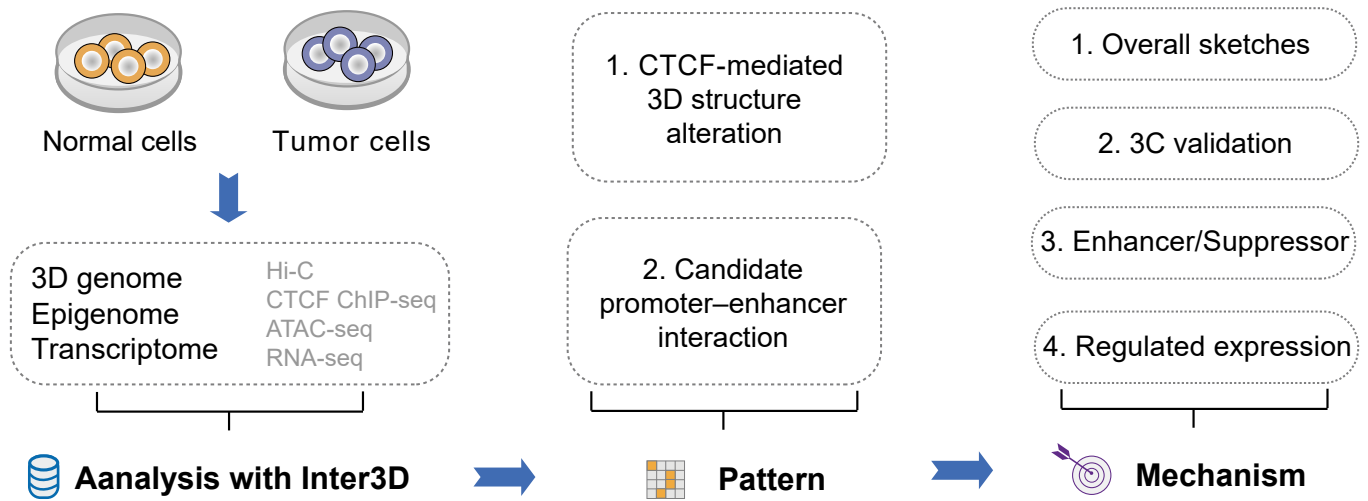**B**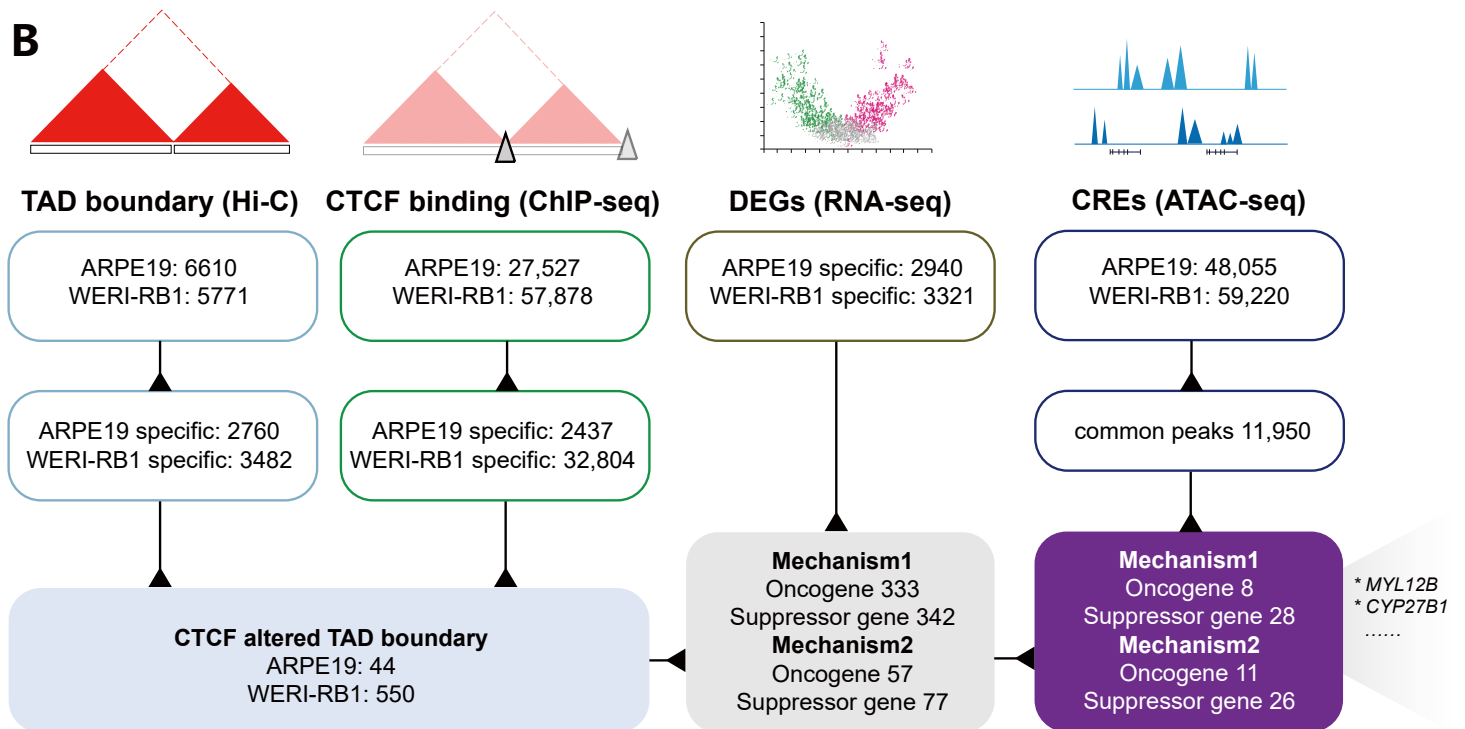

Supplement: qzae034_Supplementary_Data [file qzae034_supplementary_data.zip › Supplementary_Figure 1.pdf]

**A**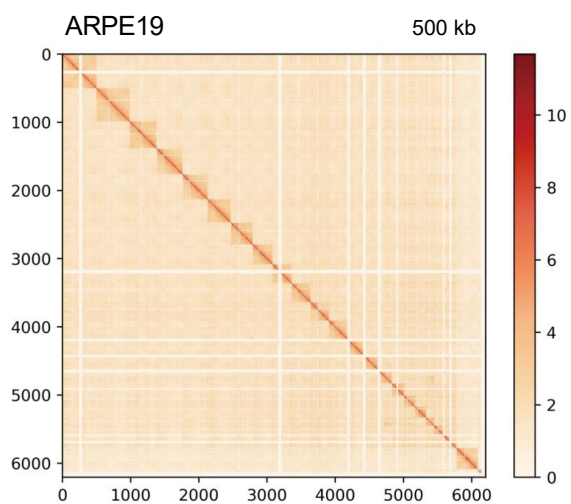**B**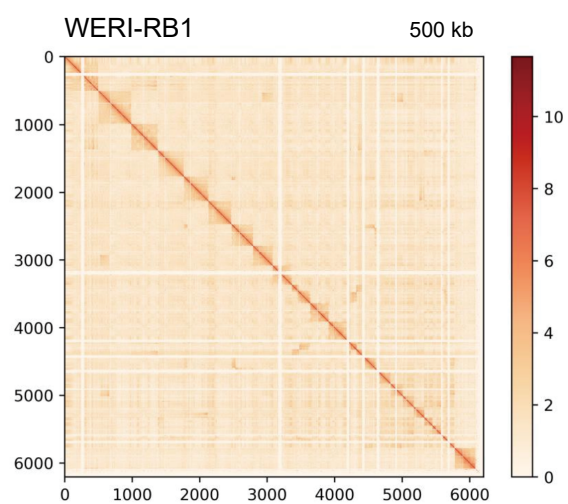**C**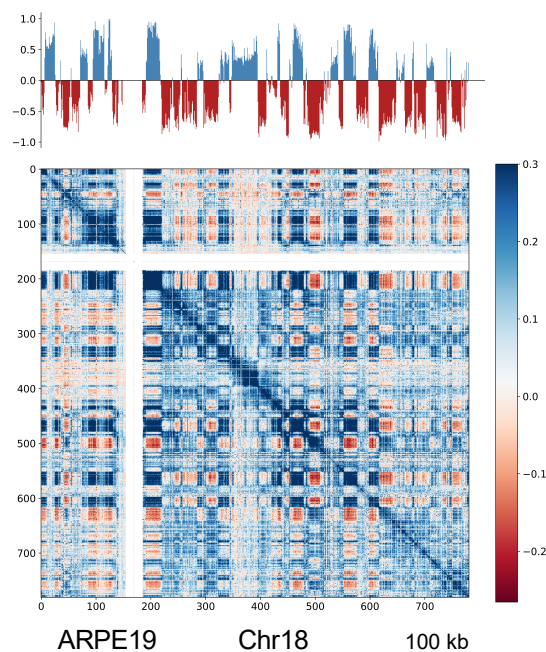**D**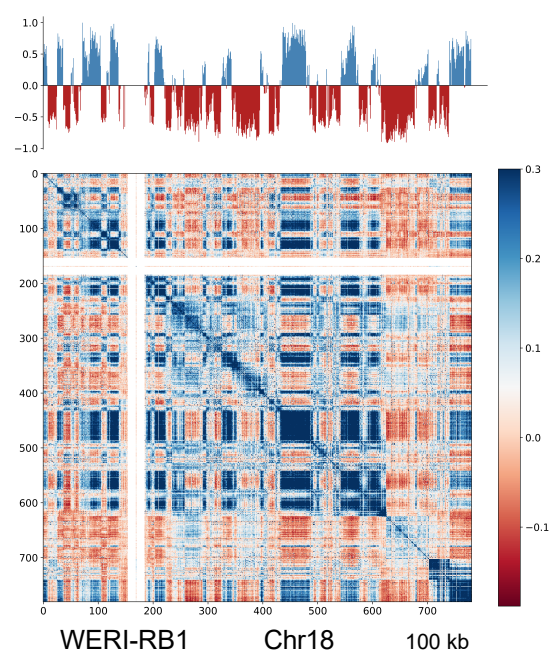**E**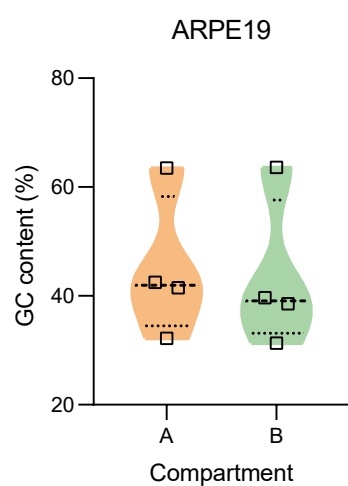**F**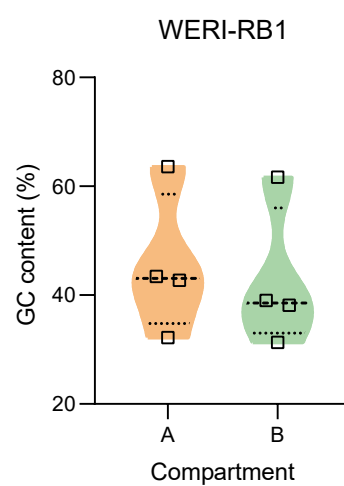

Supplement: qzae034_Supplementary_Data [file qzae034_supplementary_data.zip › Supplementary_Figure 2.pdf]
